# Progressive lysosomal membrane permeabilization induced by iron oxide nanoparticles drives hepatic cell autophagy and apoptosis

**DOI:** 10.1186/s40580-020-00228-5

**Published:** 2020-05-19

**Authors:** Kateryna Levada, Stanislav Pshenichnikov, Alexander Omelyanchik, Valeria Rodionova, Aleksey Nikitin, Alexander Savchenko, Igor Schetinin, Dmitry Zhukov, Maxim Abakumov, Alexander Majouga, Mariia Lunova, Milan Jirsa, Barbora Smolková, Mariia Uzhytchak, Alexandr Dejneka, Oleg Lunov

**Affiliations:** 1 Institute of Physics, Mathematics and Information Technology, Immanuel Kant Baltic Federal University, Kaliningrad, Russia; 2 National University of Science and Technology “MISIS”, Moscow, Russia; 3 Institute of Physics of the Czech Academy of Sciences, 18221 Prague, Czech Republic; 4 Institute for Clinical & Experimental Medicine (IKEM), Prague, Czech Republic

**Keywords:** Cytotoxicity, Apoptosis, Autophagy, Iron oxide nanoparticles, Magnetic resonance imaging

## Abstract

Iron oxide nanoparticles (IONs) are frequently used in various biomedical applications, in particular as magnetic resonance imaging contrast agents in liver imaging. Indeed, number of IONs have been withdrawn due to their poor clinical performance. Yet comprehensive understanding of their interactions with hepatocytes remains relatively limited. Here we investigated how iron oxide nanocubes (IO-cubes) and clusters of nanocubes (IO-clusters) affect distinct human hepatic cell lines. The viability of HepG2, Huh7 and Alexander cells was concentration-dependently decreased after exposure to either IO-cubes or IO-clusters. We found similar cytotoxicity levels in three cell lines triggered by both nanoparticle formulations. Our data indicate that different expression levels of Bcl-2 predispose cell death signaling mediated by nanoparticles. Both nanoparticles induced rather apoptosis than autophagy in HepG2. Contrary, IO-cubes and IO-clusters trigger distinct cell death signaling events in Alexander and Huh7 cells. Our data clarifies the mechanism by which cubic nanoparticles induce autophagic flux and the mechanism of subsequent toxicity. These findings imply that the cytotoxicity of ION-based contrast agents should be carefully considered, particularly in patients with liver diseases.

## Introduction

In last years, iron oxide nanoparticles (IONs) have been extensively studied and subsequently showed deliberate applicability in different biomedical applications, such as magnetic cell labeling for in vitro and in vivo imaging [1], guided cell delivery [2], cell manipulation [3, 4], gene/drug delivery [5, 6], studies of cellular mechanics [7, 8] and magnetic resonance imaging (MRI) [9–11]. Concomitantly with rise in usage of IONs, such particles started to be extensively recognized as a persistent environmental pollutant [12–14]. In fact, pollution-derived iron oxide nanoparticles have been found even in human brains and may represent a human health threat [15]. Taking into account that IONs are studied as potential MRI contrast agents, toxicological evaluation of IONs requires deep analysis.

Indeed, initial cytotoxicity studies showed that IONs had presumably low cytotoxicity profile and are well tolerated by the organism [16]. Furthermore, iron oxide nanoparticles have been found to be produced endogenously by bacteria [17]. These facts together created an attitude that IONs would be of a high biocompatibility and well tolerated by human body [16]. As a result, IONs were initially approved for clinical use as negative MRI contrast agents [9, 10]. However, growing body of evidence has demonstrated a range of toxic effects associated with exposure to IONs [18–27]. Thus, in recent years a number of prominent ION contrast agents have been withdrawn or discontinued [9, 10, 28, 29]. This fact clearly indicates that cytotoxic potential of IONs was overlooked. Although emerging evidence suggests that toxicity of IONs is concentration dependent and also depends on exposure time [18–27], still our knowledge about the underlying mechanisms of IONs-induced toxicity and their physiological and pathophysiological effects remains rather limited. Therefore, systematic scientific examination of possible adverse effects of IONs are of great importance.

Indeed, cytotoxicity of IONs was shown in various cell lineages, e.g. on cultured human monocytes, murine and human macrophages [18–21], on mouse glioma cells [30], human epithelial colorectal adenocarcinoma cells [31], human pancreas and kidney cells and neurons [23]. So far, only few studies elucidated cytotoxic potential of IONs on liver cell cultures [32–36]. It is worth noting here, that hepatotoxicity is one of the most common reasons for withdrawals of drugs from the market worldwide accounting for ~ 30% of such cases [37]. Therefore, in this study we investigated ION-mediated adverse effects on hepatic cell lines. Interestingly, cubic IONs showed superior MRI contract versus other shapes [38, 39]. Thus, as a particle model in our study, we utilized iron oxide nanocubes (IO-cubes) and clusters of nanocubes (IO-clusters).

Most studies decipher ROS generation and consequent oxidative stress as a major reason for ION toxicity [18–27]. Effect of reactive oxygen species (ROS) formation through IONs is poorly described in sense of effect on different cell lines and regime of administration [40]. However, a controlled toxicity process can be used for therapeutic purposes. For example, very recently an IONs-based FDA-approved drug (Ferumoxytol) was utilized for the treatment of iron deficiency anaemia in the USA and was noted as an off-label MRI contrast agent that showed antileukaemia effects [41, 42].

Recently, IONs have been reported to induce autophagy [27, 43–47]. Furthermore, recent reports suggest that nanoparticles induce autophagy together with apoptosis [48–50]. Taking into account a substantial molecular crosstalk between apoptosis and autophagic death pathway [51, 52], we hypothesized that IONs might impact on both apoptosis and autophagy. Here we demonstrate that IO-cubes and IO-clusters exposure leads to the cytotoxicity in different hepatic cell lines associated with an induction of lysosomal membrane permeabilization-driven apoptosis and autophagy. To the best of our knowledge, there is no report so far on autophagy caused by IONs in human hepatic cells.

## Experimental

### Chemicals and antibodies

Iron (III) acetylacetonate (Fe(acac)_3_; ≥ 99.9%), benzyl ether (98%), oleic acid (OA; tested according to Ph. Eur.), oleylamine (technical grade, 70%), 1,2-hexadecanediol (technical grade, 90%), ammonium acetate (99.99%), 3-(2-Pyridyl)-5,6-diphenyl-1,2,4-triazine-p,p′-disulfonic acid monosodium salt hydrate (Ferrozine; 97%), l-ascorbic acid (≥ 99.0%), α,ω-Bis[2-[(3-carboxy-1-oxopropyl)amino]ethyl]polyethylene glycol (M_r_ = 6000), N-Hydroxysuccinimide (98%), N-(3-Dimethylaminopropyl)-N′-ethylcarbodiimide hydrochloride (≥ 99.0%), potassium carbonate (K_2_CO_3_; anhydrous, ≥ 99%), sodium hydroxide (NaOH; ≥ 97.0%), dichloromethane (CH_2_Cl_2_; ≥ 99.8%), N,N-Dimethylformamide (DMF; anhydrous, 99.8%) were purchased from Sigma-Aldrich. 6-Nitrodopamine was purchased from Toronto Research Chemicals. 1-Indanecarboxylic acid (95%) was purchased from ABCR GmbH & Co. KG. Cyanine5 amine (Cy5) was purchased from Lumiprobe. Hydrochloric acid (HCl; 36%) was purchased from Sigma-Tek (Moscow, Russia). Hexane (≥ 98.0%), 2-propanol (≥ 99.8%) and nitric acid (HNO_3_; ≥ 65.0%) were purchased from Component-Reaktiv (Moscow, Russia). All reagents were used without any further purification. Ultra-pure Milli-Q water was obtained by means of Millipore Milli-Q Academic System.

The following fluorescent probes were used: LysoTracker^®^ green (200 nM) to monitor lysosomal morphology and integrity (Thermo Fisher Scientific); VAD-FMK conjugated to FITC (FITC-VAD-FMK) to detect caspase-3 activation (Abcam). To investigate mitochondrial dynamics, cells were loaded with MitoTracker^®^ green (0.5 μM; Thermo Fisher Scientific) by incubating them for 15 min. The cell-permeant fluorescent nucleic acid stain hoechst 33342 (Thermo Fisher Scientific) was used to label nucleus. The optimal incubation time for each probe was determined experimentally.

The following antibodies were used: anti-LC3A/B, dilution 1:1000 (#12741, Cell Signaling Technology); anti-RIP1, dilution 1:1000 (#ab72139, Abcam); anti-β-actin, dilution 1:2000 (#10D10, Thermo Fisher Scientific); annexin V Alexa Fluor™ 488, dilution 1:100 (#V13245, Thermo Fisher Scientific); anti-mouse-HRP, dilution 1:10,000 (#G21040, Thermo Fisher Scientific); anti-rabbit-HRP, dilution 1:10,000 (#G21234, Thermo Fisher Scientific).

### Synthesis of the iron oxide nanocubes (IO-cubes) and nanoclusters (IO-clusters)

Cubic magnetite nanoparticles were synthesized through thermal decomposition of iron(III) acetylacetonate in benzyl ether in the presence of 1,2-hexadecanediol, oleic acid and oleylamine. Briefly, the mixture of Fe(acac)_3_ (1 mmol), 1,2-hexadecanediol (8 mmol), oleic acid (16 mmol) and oleylamine (4 mmol) in benzyl ether (20 mL) were warmed up to 130 °C under argon flow and magnetic stirring and maintained for 1 h to remove trace amounts of water and oxygen. After that the mixture was heated to boiling point with a rate of 3 °C/min and kept at this temperature for 4 h. Then the solution was cooled down to the room temperature and nanoparticles were collected by centrifugation (6000 rpm, 30 min) after adding of 2-propanol (10 mL) and hexane (10 mL) and stored in dichloromethane at 4 °C.

Cluster magnetite nanoparticles were synthesized through thermal decomposition of iron(III) acetylacetonate in benzyl ether in the presence of 1,2-hexadecanediol and 1-Indanecarboxylic acid. For this Fe(acac)_3_ (2 mmol), 1,2-hexadecanediol (8 mmol) and 1-Indanecarboxylic acid (6 mmol) were dissolved in 20 mL of benzyl ether. After that the mixture was warmed up to 130 °C under argon flow and magnetic stirring and maintained for 1 h to remove trace amounts of water and oxygen. After that the mixture was heated to 210 °C with a rate about 10 °C/min and kept at this temperature for 1 h and finally the temperature of mixture was raised to 260 °C with a rate of 10 °C/min and kept at for 30 min. Then the solution was cooled down to the room temperature and nanoparticles were collected by centrifugation (6000 rpm, 30 min) after adding of 2-propanol (10 mL) and hexane (10 mL) and stored in dichloromethane at 4 °C.

To transfer the synthesized nanoparticles from nonpolar organic to aqueous medium they were modified with polyethylene glycol derivative. Briefly, α,ω-Bis[2-[(3-carboxy-1-oxopropyl)amino]ethyl]polyethylene glycol (40 mg), NHS (2 mg), EDC (3 mg), 6-Nitrodopamine (1.5 mg) and K_2_CO_3_ were mixed together in CH_2_Cl_2_ (2 mL) and DMF (1 mL). The obtained mixture was bubbled with argon for 5 min and stirred on a magnetic stirrer for 2 h. After that 1 mL of nanoparticles in CH_2_Cl_2_ with concentration 5 mg/mL was added to resulting solution and intensively stirred overnight. After that 3 mL of hexane was added to the obtained mixture and nanoparticles were collected with permanent magnet and dried with argon. Finally, magnetic nanoparticles were redispersed in 5 mL of pure deionized water and dialyzed for 24 h in dialyzing tubes with pore size 12–14 kDa to remove any impurities.

To modify the obtained nanoparticles with fluorescent dye Cy5-amine, 1 mL of water solution of α,ω-Bis[2-[(3-carboxy-1-oxopropyl)amino]ethyl]polyethylene glycol modified IO-cubes/IO-clusters (1 mg/mL) was mixed with 14 µL of EDC water solution (10 mg/mL) and 8 µL of NHS water solution (10 mg/mL) and incubated for 20 min. After that, 0.1 mL of Cy5-amine water solution ([Cy5-amine] = 1 mg/mL) was added to the reaction mixture and the solution was shaken overnight. The resulting nanoparticles modified with Cy5-amine were purified from unreacted NHS, EDC and dye molecules using PD-10 column and dialysis in dialyzing tubes with pore size 12–14 kDa.

### Physicochemical characterization of the IO-cubes and IO-clusters

Micrographs of synthesized nanoparticles were taken by transmission electron microscopy (TEM) on a JEOL JEM-1400 (120 kV) microscope. All samples were prepared by dropping of nanoparticles water dispersion onto a carbon-coated copper grid (300 mesh) and subsequently evaporating of the solvent. The average diameter of the samples and size distribution were evaluated by using ImageJ software. At least 500 nanoparticles were analyzed for each sample.

XRD patterns at room temperature were obtained using an X-ray power diffractometer DRON-4 with Co Kα radiation. The data were collected from 2θ = 20 to 120° at a scan rate 0.1° per step and 3 s per point. Qualitative phase analysis was performed by comparison of obtained spectra with PHAN database. Quantitative analysis (including crystal size evaluation by determination of coherent-scattering region) was performed using PHAN % and SPECTRUM programs developed by Physical Materials Science Department of NUST “MISiS” (modification of Rietveld method).

Magnetic hysteresis loops were obtained on “Quantum Design” Physical Property Measurement System (PPMS) equipped with vibrating sample magnetometer (VSM) with 2 mm amplitude of oscillations, 40 Hz frequency. The measurements were carried out from − 30 to 30 kOe at room temperature (300 K). Values of saturation magnetization are extracted with fitting of high-field region using law of approach to saturation (LAS) [53].

The hydrodynamic size of nanoparticle solutions was analyzed by dynamic light scattering (DLS). The measurements were performed on Zetasiser Nano ZS device. The nanoparticles concentration in each sample was 0.5 mg/mL.

### Cell culture

In this study we used three well-established hepatic cells lines: Huh7 (Japanese Collection of Research Bioresources, JCRB), Alexander (PLC/PRF/5, American Type Culture Collection, ATCC, Manassas, VA, US) and HepG2 (American Type Culture Collection, ATCC, Manassas, VA, US). Cells were cultured according to supplier guidelines in EMEM medium (ATCC) supplemented with 10% fetal bovine serum (FBS, Thermo Fisher Scientific). Cultures were kept in a humidified 5% CO_2_ atmosphere at 37 °C.

### Cell viability assay

Cells were seeded onto 96-well plates at a density of 10 000 cells per well. Following seeding, cells were stimulated with distinct concentrations of two types of particles for 48 h time period. The alamarBlue viability assay (Thermo Fisher Scientific) was utilized to assess cytotoxicity of the IO-cubes and IO-clusters [54, 55]. Briefly, alamarBlue reagent was added to each well and incubated for 2 h at 37 °C according to the manufacturer’s instructions. Microplate reader SpectraFluor Plus (TECAN, Mannedorf, Switzerland) was used to assess fluorescence increase (excitation between 530 and 560; emission at 590 nm). Three independent experiments were performed for each measurement. Readings were done in triplicates.

### Apoptosis assay

We utilized a Dead Cell Apoptosis Kit (Thermo Fisher Scientific, Waltham, MA) for the analysis of early sings of apoptosis. Following nanoparticle treatment, cells were stained with Dead Cell Apoptosis Kit according to the manufacturer’s instructions. Phosphatidylserine expression was analyzed by annexin V staining. Membrane permeability was assessed by propidium iodide labeling. Hoechst 33342 served as nucleus stain. Following staining, 4% paraformaldehyde fixation for 10 min at room temperature was performed. Treatment with 2 µM staurosporine for 3 h served as a positive control. Fluorescence images were recorded with epifluorescent microscope IM-2FL (Optika Microscopes, Ponteranica, Italy). ImageJ software was used for image processing and fluorescent micrograph quantification.

### Caspase-3 activity assay

To verify apoptosis pathway, we additionally assessed another specific hallmark—activity of caspase-3. We utilized VAD-FMK conjugated to FITC (FITC-VAD-FMK) caspase-3 inhibitor. This inhibitor is cell permeable, nontoxic, and irreversibly binds to activated caspases in apoptotic cells. Following nanoparticle treatment, cells were loaded with FITC-VAD-FMK (Abcam) in accordance with guidelines of the manufacturer. Afterwards, stained cells were imaged using high-resolution spinning disk confocal microscopy IXplore SpinSR (Olympus). Fluorescence intensity was measured using ImageJ software (NIH). As a positive control, cells were treated 3 h with 2 μM staurosporine.

### Lysosomal stability assessment

After nanoparticle treatment cells were stained with LysoTracker green (Thermo Fisher Scientific). This dye partitions to acidic vacuoles and its fluorescent intensity reflects accumulation in such structures [56]. Upon lysosomal membrane permeabilization, there is a loss of accumulated LysoTracker green fluorescence signal. One can detect such changes fluorometrically or alternatively using confocal microscopy [56]. We utilized spinning disk confocal microscopy IXplore SpinSR (Olympus) to estimate the decrease in fluorescence intensity of LysoTracker green. Fluorescence intensity was measured using ImageJ software (NIH). As a positive control, cells were treated with 20% ethanol for 20 min.

### Cell extracts and immunoblot analysis

We performed immunoblot analysis utilizing previously described procedure [21, 55, 57, 58]. Whole cell lysate was prepared using lysis buffer RIPA. SDS-PAGE electrophoresis was utilized to separate proteins according to their molecular weight, then proteins were transferred to PVDF membranes. The membranes were blocked with 5% (w/v) fat free dried milk for 1 h. Following blocking, membranes were stained with various specific primary antibodies at 4 °C overnight and detected as described [21, 55, 57]. All antibodies used in the study are summarized in 2.1 section.

### Confocal microscopy

For high quality confocal images, we used brand new high-resolution spinning disk confocal system IXplore SpinSR (Olympus) [54, 59]. This technique was utilized to assess in great details the morphological changes of cells upon nanoparticle treatment. Fluorescence images were taken with the acquisition software cellSens (Olympus). ImageJ software (NIH) was used for image processing and quantification.

### Fluorescent image processing and quantification

For quantitative assessment of taken fluorescent images corrected total cell fluorescence (CTCF) methodology was used [2, 60]. CTCF was measured in ImageJ software (NIH). Fluorescence intensity can be easily assessed by normalization of CTCF of the full area of interest to average fluorescence of a single cell. Previously described and validated method was used for calculation of the net average CTCF intensity of a pixel in the region [2, 60]. The region placed in an area without fluorescent objects was used for background subtraction. CTCF was determined as the sum of pixel intensity for a single image with the subtracted average signal per pixel for a region selected as the background. Averages of normalized intensity values of at least 15 morphologically identical cells were calculated to determine the mean fluorescence of a single cell.

### Immunofluorescence staining

Cells were seeded on μ-Slides (Ibidi, Martinsried); then, incubated with cell culture media (EMEM 10% FBS) containing different types of nanoparticles either IO-cubes or IO-clusters (both 100 μg/mL) for 24 h at 37 °C and 5% CO_2_. Afterwards, cells were fixed in 4% paraformaldehyde in PBS for 10 min, permeabilized in 0.5% TritonX 100 in PBS for 20 min, then labeled with anti-LC3A/B antibody, dilution 1:100 (#12741, Cell Signaling Technology) followed by labeling with anti-rabbit Alexa Fluor 488 conjugated antibody, dilution 1:1000 (#A-11008, Thermo Fisher Scientific). Hoechst 33342 (Thermo Fisher Scientific) was used to label nucleus. Fixed cells were imaged using a spinning disk confocal system IXplore SpinSR (Olympus).

### LC3 real-time PCR

Cells were incubated with different types of nanoparticles either IO-cubes or IO-clusters (both 100 μg/mL) for 24 h at 37 °C and 5% CO_2_. Afterwards, total RNA from cells was isolated using RNeasy mini kit (cat. 74106, Qiagen) with further DNA removal by using DNase I digestion and RNeasy Kit (cat. 79254, Qiagen). Quality and quantity of achieved RNA was detected using NanoDrop 8000 (Thermo Fisher) and 2 µg of RNA was transcribed into cDNA with a Maxima H Minus First Strand cDNA Synthesis Kit (cat. K1682, Thermo Fisher).

Quantitative real-time PCR was performed by using Viia7 (Applied Biosystems, Real Time PCR system), Fast Advanced TaqMan Gene expression Master mix (cat. 4444557, Thermo Fisher) and specific Bio-Rad PrimePCR™ Probe Assays (*MAB1LC3B*, *GAPDH*). Total RNA input was 20 ng per reaction. Samples were analyzed in quadruplicates per each group. *GAPDH* Probe Assay was used as an internal control. Data were analyzed using Excel and MaxStat Pro 3.6 programs. Expression of target gene was normalized to *GAPDH* expression by using the 2^−ΔΔCT^ method as described in [61].

### Statistical analysis

Quantitative results are present as mean ± SEM. The statistical significance of differences between the groups was determined using ANOVA Newman-Keuls test. All statistical analyses were performed using MaxStat Pro 3.6. Statistical significance was identified if the tested *p* value was smaller than 0.05 (*), 0.01 (**) or 0.001 (***). When multiple pairwise comparisons were performed, the Bonferroni correction was used to adjust the significance level.

For quantitative fluorescence microscopy analysis (analysis of lysosomal size, integrity, caspase-3 activity) rigorously defined guidelines for accuracy and precision quantification were used [62]. The sample size determination was based on a statistical method described in [63], which determines sample size for 95% confidence level and 0.8 statistical power equal to 15. Therefore, n = 15 cells were used in quantification.

## Results

### Characterization of the nanoparticles

**Fig. 1:**
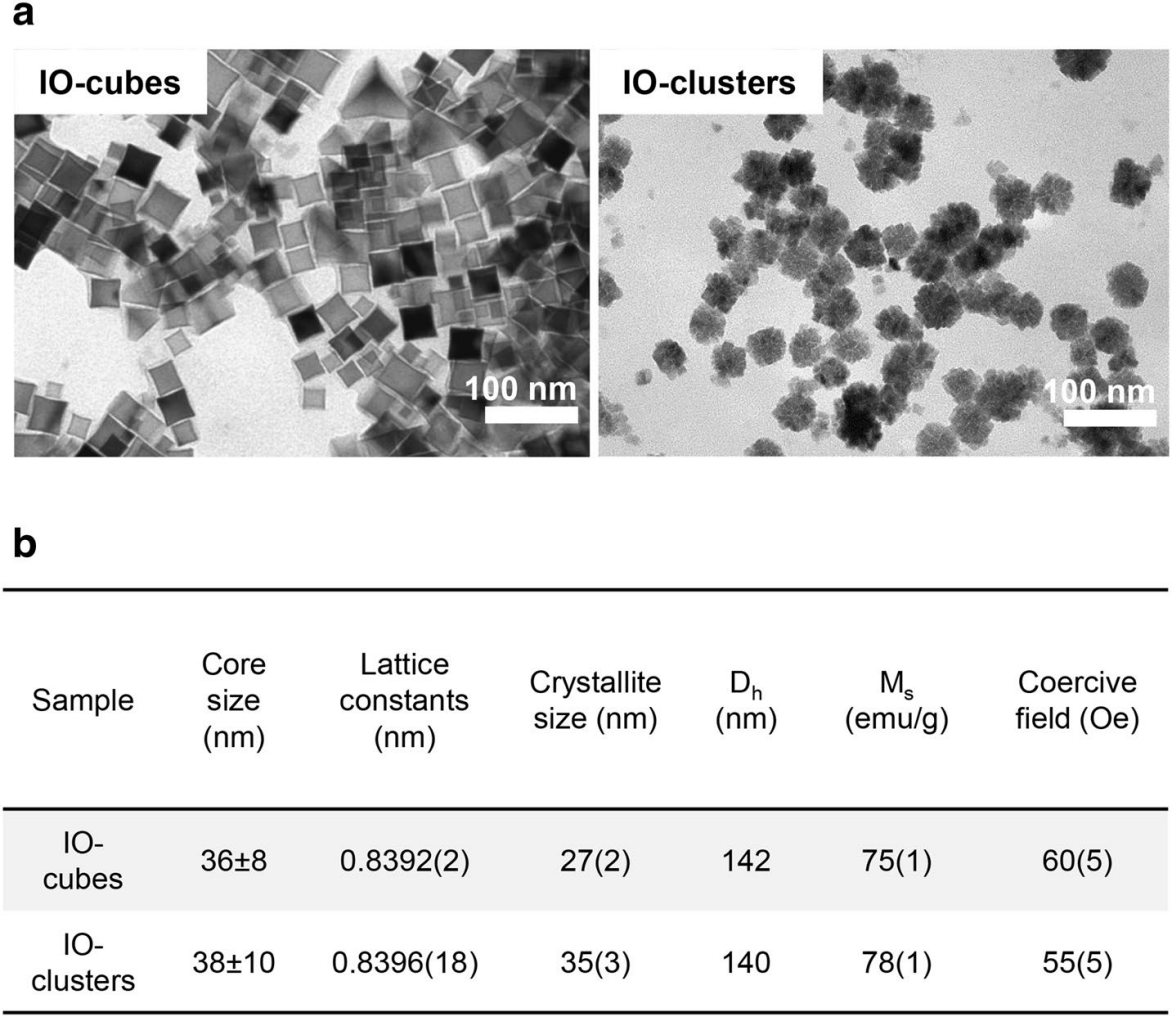
Physicochemical characterization of the iron oxide nanocubes (IO-cubes) and nanoclusters (IO-clusters). **a** Transmission electron micrographs of the iron core of the nanoparticles. **b** Physicochemical properties of IO-cubes and IO-clusters (*D*_*h*_ hydrodynamic diameter, *M*_*s*_ saturation magnetization)

**Fig. 2:**
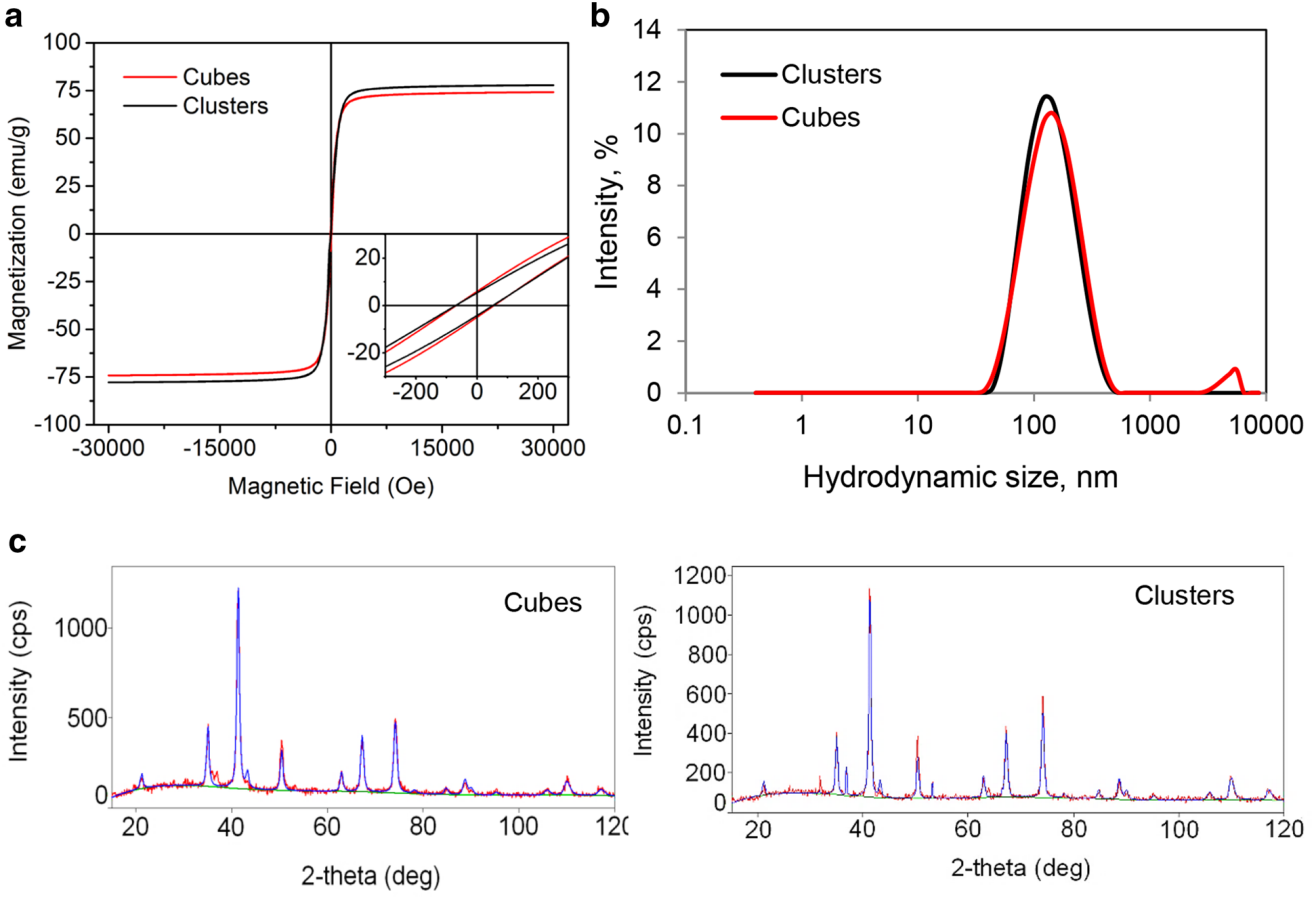
Physicochemical characterization of the iron oxide nanocubes (IO-cubes) and nanoclusters (IO-clusters). **a** Room temperature magnetization curves of IO-cubes and IO-clusters using magnetic field range from − 30 to 30 kOe. **b** Hydrodynamic diameters of IO-cubes and IO-clusters in PBS after surface modification with PEG as measured by dynamic light scattering utilizing Zetasiser Nano ZS. **c** XRD patterns of IO-cubes and IO-clusters with the indexation of the Bragg peaks to an inverse spinel structure

IO-cubes and IO-clusters were synthetized and functionalized as previously described [64, 65]. The physicochemical properties of the IO-cubes and IO-clusters investigated in this study are summarized in Figs. 1 and 2. Transmission electron microscopy of both preparations revealed the same mean size of an iron oxide core of about 36 and 38 nm for IO-cubes and IO-clusters respectively (Fig. 1a and Additional file 1: Figure S1). Accordingly, dynamic laser light scattering analysis in aqueous environment showed mean hydrodynamic diameters of about 140 nm for both IO-cubes and IO-clusters (Figs. 1b, 2b). The magnetization curves were similar for IO-cubes and IO-clusters (Fig. 2a). Finally, XRD was used to confirm IO-cubes and IO-clusters structure and phase composition (Fig. 2c).

It is worth noting here, that one of the widest areas of biomedical application of iron oxide nanoparticles is an MRI contrasting [28, 66]. Indeed, it was found that the size of nanoparticles between 50 and 200 nm seems to be the most effective for cellular uptake and MRI imaging applications [10, 28, 66, 67]. Particles having size comparable with the diameter of liver sinusoidal fenestrations (up to 150–200 nm) have been shown to extravasate into the space of Disse and interact directly with hepatocytes [66, 68]. Thus, these findings taken together make particles of ~ 200 nm size a great candidate to study the molecular basis of nanoparticle–hepatocyte interaction.

### Treatment with IO-cubes or IO-clusters induces acute toxicity in hepatic cells

Overall, iron oxide nanoparticles (IONs) showed to be an excellent MRI contrast agents [9, 10]. However, a number of ION contrast agents have been withdrawn due to their poor clinical contrast performance and/or safety concerns [9, 10, 28, 29]. Many studies so far have identified significant acute cytotoxicity of IONs on cultured human monocytes, murine and human macrophages [18–21], on mouse glioma cells [30], human epithelial colorectal adenocarcinoma cells [31], human pancreas, kidney cells and neurons [23]. It becomes evident that there exists a correlation between the mechanism of toxicity of IONs and major physicochemical factors responsible for in vitro/in vivo toxicity [23]. However, the underlying mechanisms responsible for the toxic actions of nanoparticles are still not clear. This prompted us to focus on the subcellular mechanisms of IONs cytotoxicity in human hepatic cells.

**Fig. 3:**
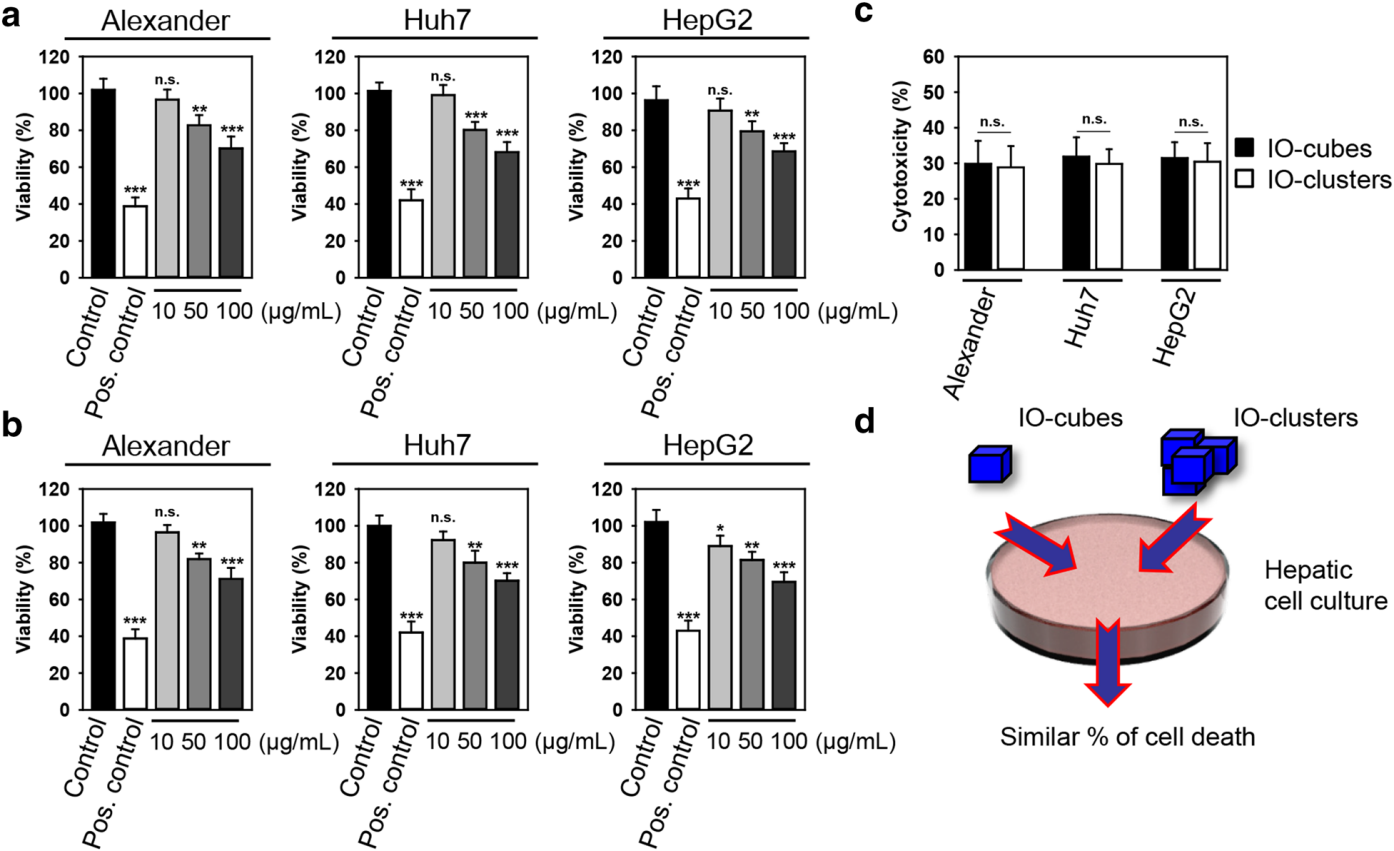
Viability studies. Alexander, HepG2 and Huh7 cells were treated with indicated concentrations of either IO-cubes (**a**) or IO-clusters (**b**) for 48 h. The alamarBlue cell viability assay was used to assess degree of cellular viability. The data were normalized to control values (no particle exposure), which were set as 100% cell viability. Control cells were untreated. As a positive control, cells were treated with 20% ethanol for 60 min. Data are expressed as mean ± SEM (n = 3), **p < 0.01, ***p < 0.001. **c** Cell viability comparison among different hepatic cell lines as detected by the alamarBlue cell viability assay. Cells were treated with 100 µg/mL of either IO-cubes or IO-clusters. **d** IO-cubes and IO-clusters reduce the viability of human hepatic cell lines to a similar rate

The viability of closely related hepatic cell lines (HepG2, Huh7 and Alexander cells) was concentration-dependently decreased after 48 h exposure to either IO-cubes or IO-clusters (Fig. 3a, b). Interestingly, both particle types decreased the viability of all three cell lines by ~ 30% *p* < 0.001 (Fig. 3c). Similar cell death rate triggered by distinct iron oxide nanoparticle formulations in three cell lines (Fig. 3c, d) stimulated us to search for detailed mechanism of nanoparticle toxicity.

**Fig. 4:**
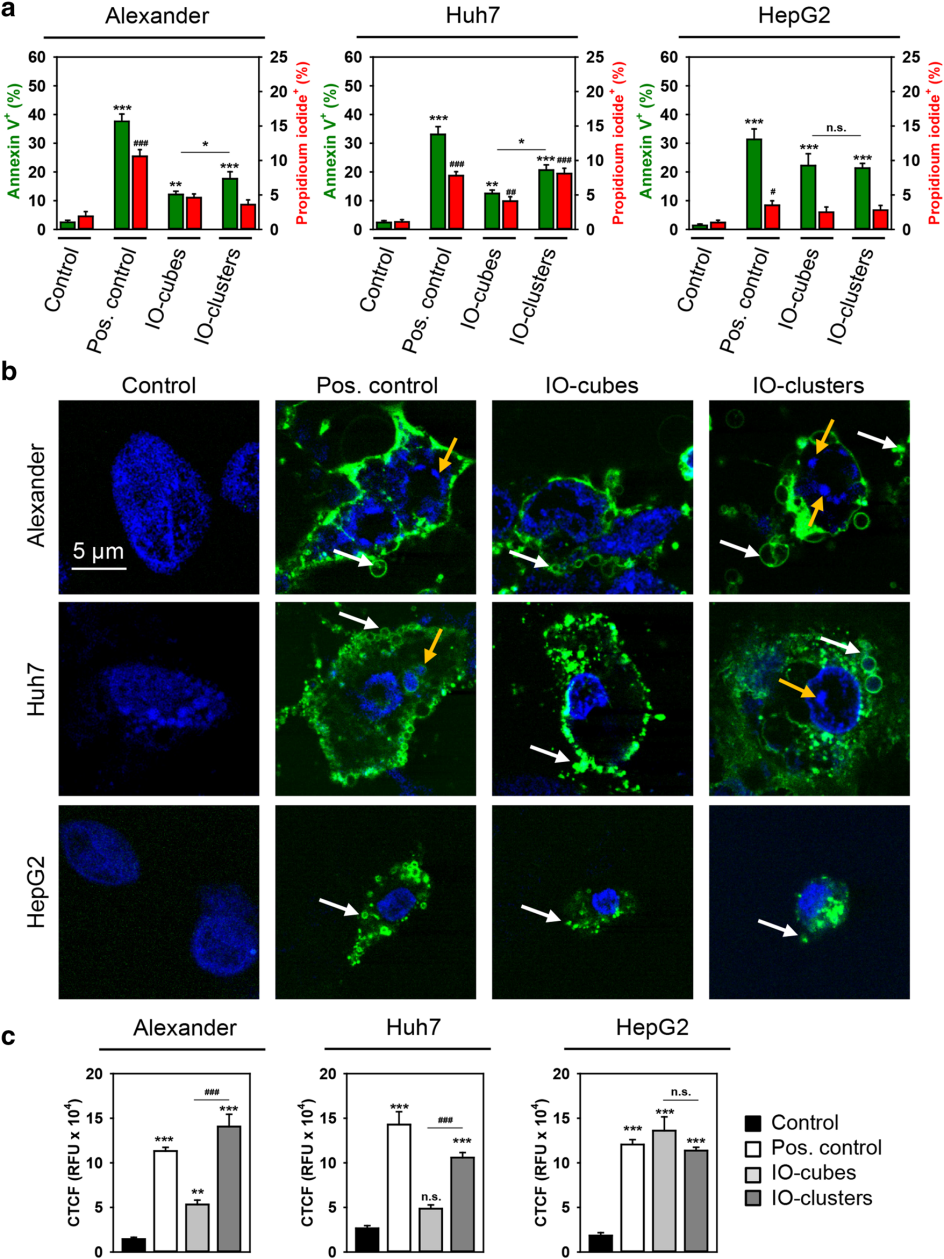
Analysis of apoptotic cell death upon treatment with IO-cubes and IO-clusters. **a** Cells were stimulated with IO-cubes or IO-clusters (100 µg/mL) for 24 h and labeled with annexin V—green dye, propidium iodide—red dye, hoechst 33342 nuclear stain—blue. Labeled cells were imaged with epi-fluorescence microscopy. ImageJ software (NIH) was used for calculation of annexin V and propidium iodide positive cells. Data are expressed as mean ± SEM (n = 3), *p < 0.05, **p < 0.01, ***p < 0.001, ^#^*p* < 0.05, ^*##*^*p* < 0.01, ^###^*p* < 0.001. Cells treated with 2 μM staurosporine for 3 h served as a positive control. **b** Cells were stimulated with IO-cubes or IO-clusters (100 µg/mL) for 24 h and then labeled with hoechst 33342 nuclear stain (blue) and annexin V (green). Yellow arrows indicate nuclear fragmentation; white arrows – blebbing of cytoplasmic membranes. Cells treated with 2 μM staurosporine for 3 h served as a positive control. Labeled cells were then imaged using high-resolution spinning disk confocal microscopy (Spin SR, Olympus). **c** Caspase-3 activation in hepatic cell lines. Alexander, HepG2 and Huh7 cells were stimulated with IO-cubes or IO-clusters (100 µg/mL) for 24 h, and incubated with fluorescein-conjugated pan-caspase inhibitor (VAD-FMK). Following the staining, cells were analyzed using a spinning disk confocal microscopy. Quantification of fluorescence intensities was performed in ImageJ (NIH) software. Data are expressed as mean ± SEM (n = 3), **p < 0.01, ***p < 0.001, ^##^*p* < 0.01, ^###^*p* < 0.001. Cells treated with 2 μM staurosporine for 3 h served as a positive control

Exposure of all three hepatic cell lines to either IO-cubes or IO-clusters for only 24 h induced early signs of apoptosis [69, 70]. We detected translocation of phosphatidylserine to the outer cell membrane leaflet utilizing annexin V labeling (Fig. 4a, b). Importantly, there was no concomitant increase in membrane permeability in Alexander and HepG2 cells (Fig. 4a, b and Additional file 1: Figures S2–S4). Furthermore, high-resolution confocal microscopy clearly showed that cells treated with IO-clusters displayed the distinctive morphological changes of apoptosis [71], such as chromatin, cell shrinkage, and nuclear fragmentation as well as blebbing of cytoplasmic membranes (Fig. 4b). Assessment of caspase-3 activity showed that, indeed, treatment with IO-clusters induced massive caspase-3 activation in HepG2, Huh7 and Alexander cells (Fig. 4c). This constellation suggested apoptotic cell death in all three cell lines initiated by IO-clusters. Contrary, treatment with IO-cubes resulted in minor or non-significant elevation of caspase-3 activity in Alexander and Huh7 cells (Fig. 4c). Taken together with annexin V/propidium iodide staining, these data imply that IO-cubes trigger cell death district from apoptosis in Alexander and Huh7 cells. Interestingly, in HepG2 both formulations of nanoparticles induced massive caspase-3 activation (Fig. 4c).

### IO-cubes trigger autophagic flux in Huh7 and Alexander cells

**Fig. 5:**
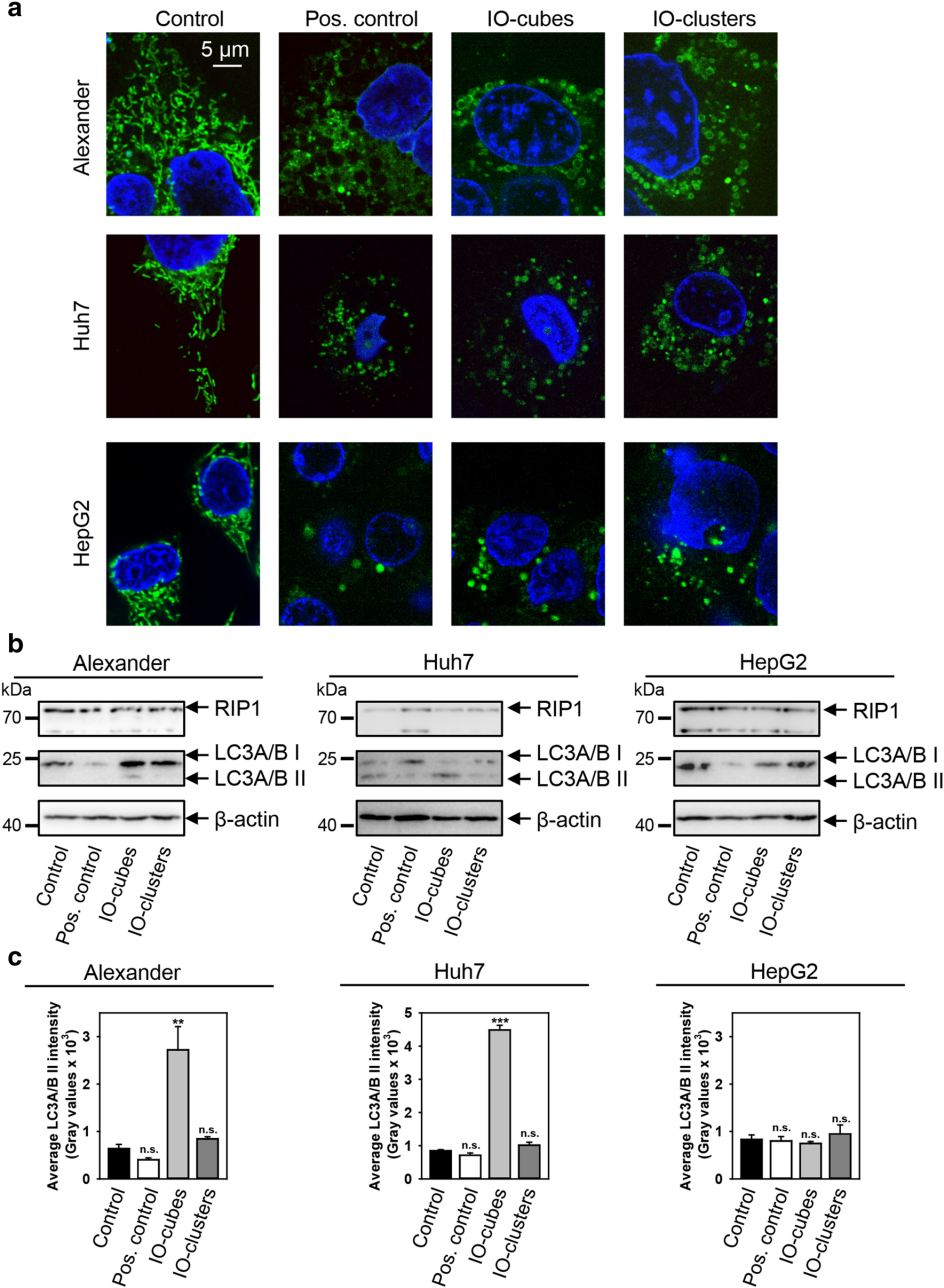
**a** Alteration of mitochondrial morphology by IO-cubes and IO-clusters treatment. Cells were stimulated with IO-cubes or IO-clusters (100 µg/mL) for 24 h and labeled with MitoTracker^®^ green. Treatment with 20% ethanol for 20 min served as a positive control. Nuclei were labelled with hoechst 33342 nuclear stain (blue). Labeled cells were then imaged using high-resolution spinning disk confocal microscopy (Spin SR, Olympus). **b** Cells were stimulated with IO-cubes or IO-clusters (100 µg/mL) for 24 h and analyzed by Western immunoblotting. Actin-control of equal protein loading. Cells treated with 2 μM staurosporine for 3 h served as a positive control. **c** Densitometric quantification of membrane-bound lipidated form of LC3. Average band intensity after Western blotting (**b**). Cells were treated as in **b**. Data are expressed as mean ± SEM (n = 3), ***p < 0.001, **p < 0.01

Our group and others have shown that ION cytotoxicity is caused by oxidative stress via redox cycling and reactive oxygen species (ROS) generation, that results in lipid peroxidation and DNA damage [20–25]. Mitochondria represents a major source of intracellular ROS [72]. Furthermore, mitochondrial dysfunction has been associated with different cell death signaling ranging from necrosis to apoptosis [73–75]. Recently, mitochondria have been identified as a novel subcellular target of ION mediated cytotoxicity [76]. It is worth noting, that analysis of the impact of ION treatment on mitochondrial activity in hepatic cells is still fragmented [23]. Therefore, we investigated whether IO-cubes or IO-clusters treatment would affect mitochondrial function. We analyzed how IO-cubes or IO-clusters treatment may affect the mitochondrial dynamics utilizing high-resolution confocal microscopy. Mitochondrial morphology in Alexander, HepG2 and Huh7 cells was visualized using MitoTracker^®^ Green labeling (Fig. 5a and Additional file 1: Figure S5). Indeed, ROS-induced oxidative stress is accompanied by mitochondrial fragmentation and fission [77, 78]. Moreover, excessive mitochondrial fragmentation is recognized as a hallmark of mitochondrial dysfunction [77, 78]. Indeed, we observed a marked increase in mitochondrial circularization in all three cell lines treated with both IO-cubes and IO-clusters compared to the controls as revealed by microscopy (Fig. 5a).

IONs have been reported as a novel class of autophagy inducers [27, 43, 44, 47, 79]. Furthermore, mitochondrial function and dysfunction have emerged as key factors in autophagy regulation [80]. Thus, we hypothesized that the perturbations in autophagic flux may explain differences in apoptosis triggering by IO-cubes and IO-clusters in distinct hepatic cell lines.

**Fig. 6:**
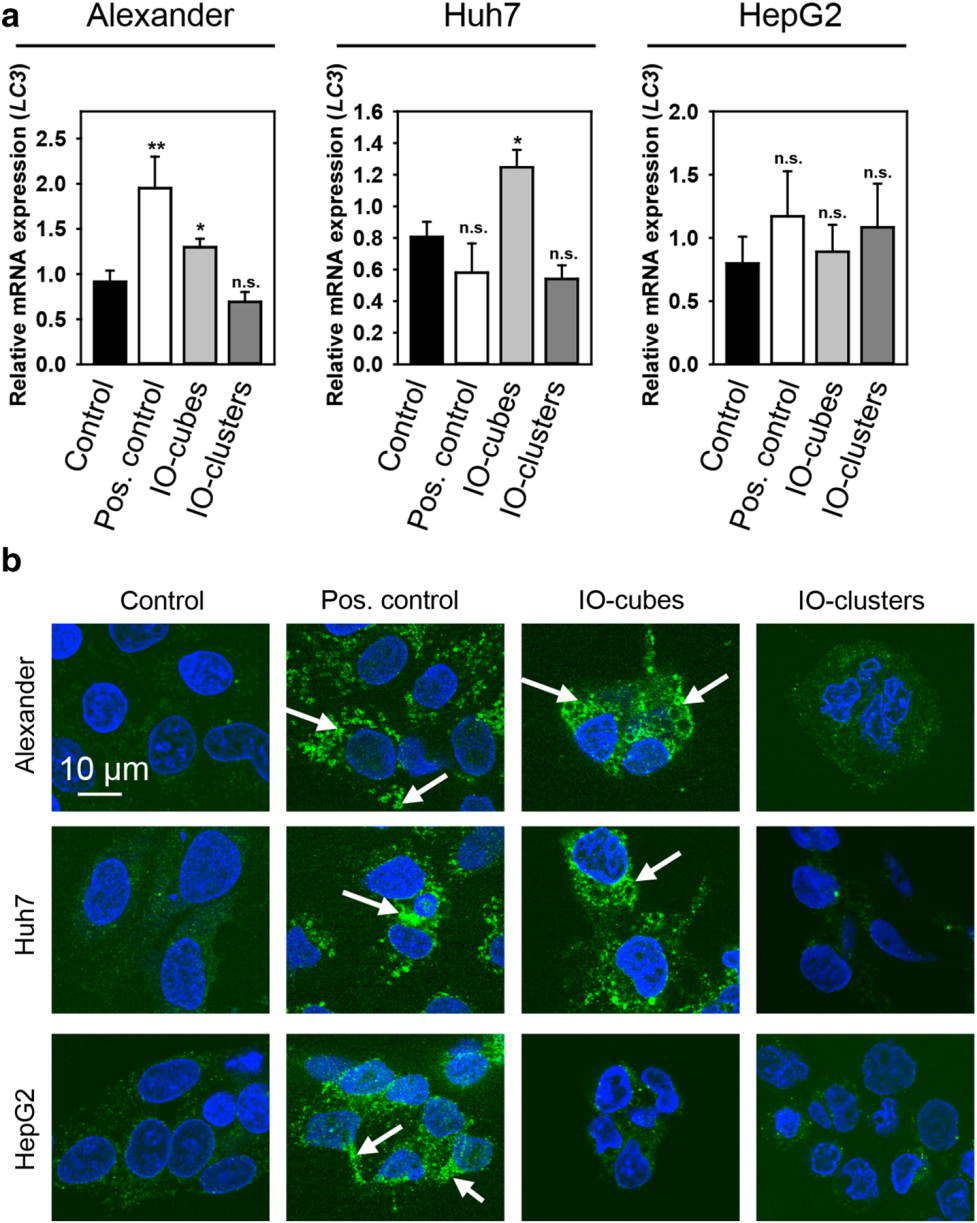
Monitoring autophagy in cells stimulated with IO-cubes or IO-clusters. **a** Cells were stimulated with IO-cubes or IO-clusters (100 µg/mL) for 24 h The relative gene expression was normalized to GAPDH expression, and calculated using the 2^−ΔΔCT^ method. Cells treated with 2 μM staurosporine for 3 h served as a positive control. Results are mean ± SEM, **p < 0.01, *p < 0.05. **b** Formation of LC3-positive puncta upon nanoparticle treatment. Cells were stimulated with IO-cubes or IO-clusters (100 µg/mL) for 24 h, fixed, permeabilized and labeled with LC3A/B (green). Nuclei were labelled with hoechst 33342 nuclear stain (blue). Labeled cells were then imaged using high-resolution spinning disk confocal microscopy (Spin SR, Olympus). White arrows indicate accumulation of autophagosomes. Positive control—serum starvation for 12 (Alexander, Huh7) and 14 (HepG2) h

To verify whether IO-cubes and IO-clusters were involved in autophagosome formation, we examined lipidation of LC3 protein. Formation of a phosphatidylethanolamine conjugated protein is a reliable indicator of autophagy [81]. As shown in Fig. 5b, IO-cubes obviously induced endogenous LC3-II transformation in Alexander and Huh7 cells, but not in HepG2 cells. Interestingly, IO-clusters had no effect on LC3 lipidation (Fig. 5b). In order to confirm initiation of autophagic flux by IO-cubes and to exclude necroptotic crosstalk, we checked an indicator of necroptosis RIP1. Indeed, neither IO-cubes nor IO-clusters showed any significant effect on RIP1 expression in all three cell lines (Fig. 5b). Consistently, densitometric analysis of western blots confirmed LC3 lipidation in Alexander and Huh7 cells, but not in HepG2 cells (Fig. 5c). Further, we analyzed *LC3* gene expression level by real time PCR (Fig. 6a). *LC3* gene expression analysis confirmed western blot data, showing LC3 gene upregulation in Alexander and Huh7 cells, but not in HepG2 cells, upon IO-cubes treatment (Fig. 6a). However, autophagy mRNA expression levels are criticized as not appropriate indicators for monitoring autophagy [82]. Therefore, to further proof execution of autophagy by IO-cubes, we assessed LC3 puncta formation, a widely used marker for autophagosomes [82]. Immunofluorescence analysis revealed massive formation of autophagosomes in Alexander and Huh7 cells, but not in HepG2 cells, upon IO-cubes treatment (Fig. 6b).

Taking together the cytotoxicity data (Fig. 3) with annexin V/propidium iodide staining (Fig. 4a, b), LC3-II transformation (Fig. 5b), LC3 gene expression (Fig. 6a) and autophagosome formation (Fig. 6b), one can clearly say that IO-cubes induce autophagic death in Alexander and Huh7 cells. Contrary, IO-clusters trigger apoptosis in Alexander and Huh7 cells. Interestingly, treatment of HepG2 with either IO-cubes or IO-clusters leads in both cases to apoptosis. Indeed, HepG2 cells show high levels of Bcl-2 in comparison with Alexander and Huh7 cells [54, 55, 83, 84]. In fact, Bcl-2 is known to block autophagy [85–87]. Thus, it may explain why both nanoparticles induce rather apoptosis than autophagy in HepG2 cells.

### Progressive lysosomal membrane permeabilization induced by IO-cubes and IO-clusters

Still we couldn’t explain why chemically similar IO-cubes and IO-clusters trigger different cell death responses in Alexander and Huh7 cells. Mounting evidence indicates that ION-induced autophagy starts at the lysosomal level [27, 43, 44, 47, 79]. Furthermore, nanoparticles have been showed to trigger lysosomal membrane permeabilization (LMP) in a progressive manner [88]. Such LMP progressively aggravates with time leading to switching between autophagy and apoptosis [88].

**Fig. 7:**
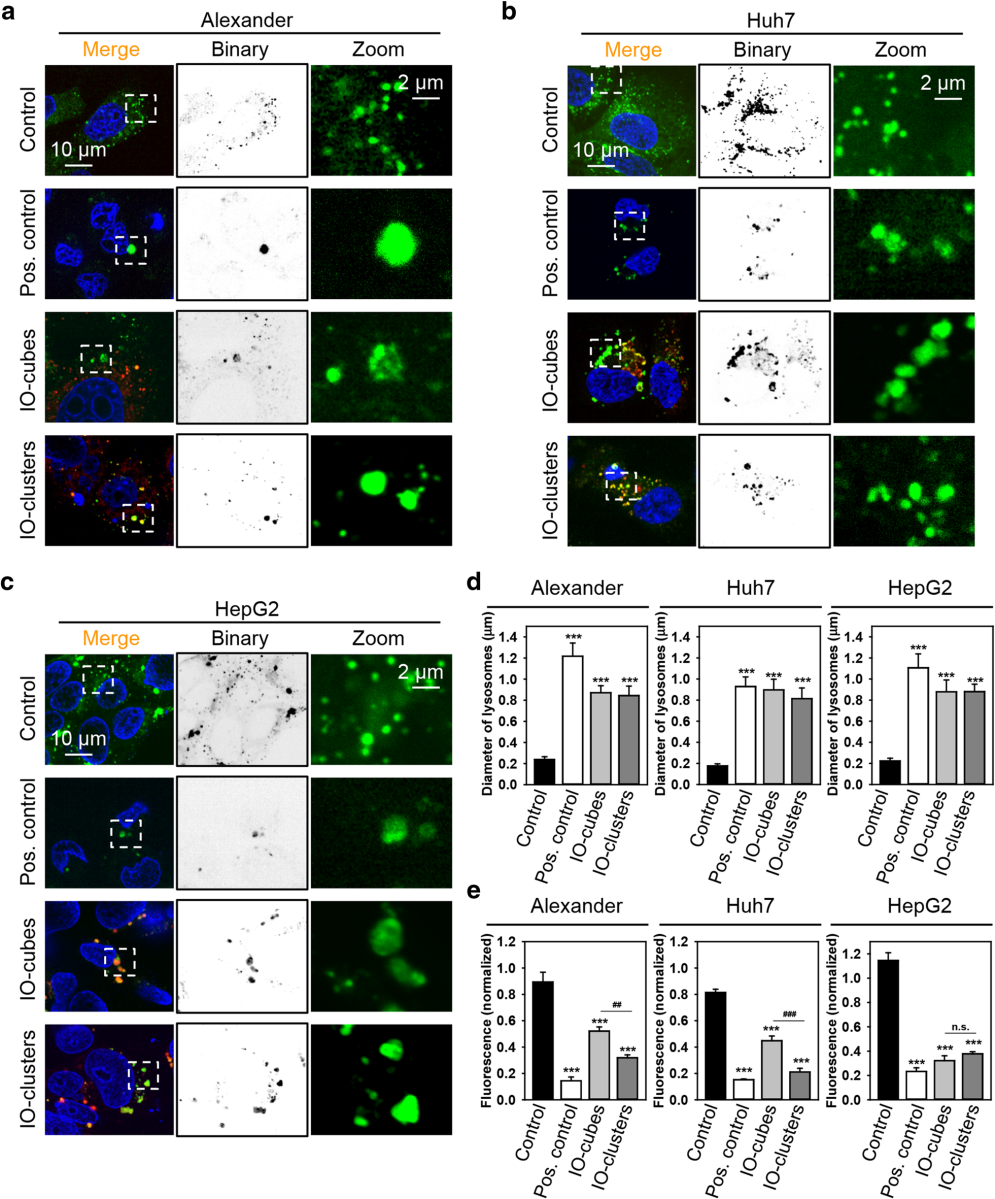
IO-cubes and IO-clusters treatment affects lysosomal integrity. Alexander (**a**), HepG2 (**b**) and Huh7 (**c**) cells were treated with fluorescently labeled (red) IO-cubes or IO-clusters (100 µg/mL) for 24 h and stained with LysoTracker (green), yellow indicates colocalization of fluorescently labeled nanoparticles with lysosomes. Positive control—20% ethanol for 20 min. Nuclei were labelled with hoechst 33342 nuclear stain (blue). Labeled cells were then imaged using high-resolution spinning disk confocal microscopy (Spin SR, Olympus). **d** Assessment of the lysosomal size upon IO-cubes or IO-clusters (100 µg/mL) uptake. Labeled cells were then imaged by confocal microscopy as in **a**–**c**, and images were quantified using ImageJ software (NIH). Quantifications performed using ImageJ are presented as means of n = 15 cells. ***p < 0.001 denote significant differences respect to control (no particle treatment). Positive control—20% ethanol for 20 min. **e** Alexander, HepG2 and Huh7 cells were exposed to IO-cubes or IO-clusters (100 µg/mL), then stained with LysoTracker and analyzed by laser scanning confocal microscopy, as described in **a**–**c**. Fluorescence intensities were analyzed with ImageJ (NIH). Data are expressed as mean ± SEM (n = 3), ***p < 0.001, ^##^*p* < 0.01, ^###^*p* < 0.001. As a positive control, cells were treated with 20% ethanol for 20 min

Therefore, we hypothesized that IO-cubes and IO-clusters may distinctly induce LMP leading to differential cell death outcomes. We analyzed whether IO-cubes and IO-clusters treatment results in distinct lysosomal destabilization in hepatic cell lines. Indeed, treatment with both IO-cubes and IO-clusters led to formation of large swollen lysosomes in all cell lines, indicating lysosomal destabilization (Fig. 7a–d and Additional file 1: Figures S6–S8). However, IO-clusters induced progressively higher LMP in comparison with IO-cubes in Alexander and Huh7 cells, as evident from LysoTracker fluorescent intensity assessment (Fig. 7e). Interestingly, IO-cubes and IO-clusters treatment showed no significant difference in extent of LMP in HepG2 cells (Fig. 7e). These data imply that IO-cubes and IO-clusters induce LMP in a progressive manner in Alexander and Huh7 cells, resulting in either autophagic death or apoptosis. Contrary, IO-cubes and IO-clusters trigger similar level of LMP in HepG2 leading to only apoptotic death.

## Discussion

Numerous studies have shown acute cytotoxicity of IONs on a number of cultured human cell lines [18–21, 23, 30, 31]. However, only few focused on liver-derived cells [32–35]. Taking into account that hepatocytes perform variety of metabolic processes, it is very important to investigate whether IONs also have unsuspected adverse effects on hepatocytes. Here we have shown that 48 h treatment with a diagnostically relevant dose of the IO-cubes and IO-clusters IONs leads to acute toxicity in hepatic cells (Fig. 3).

It is worth mentioning here, that long-circulating nanoparticles may leak preferentially into tumor through the permeable vasculature accumulating in tumor tissue [89, 90]. Such type of passive targeting is known as the enhanced permeability and retention (EPR) effect [89, 90]. The EPR effect creates a basis for passive nanosized drug delivery [90]. However, EPR effects are relatively low, leading to less than a twofold increase in delivery [89, 90]. Interesting study utilizing a meta-analysis of pre-clinical data on nanoparticle delivery to tumors showed that median delivery is still relatively low of about 0.7% of the injected dose [91]. Indeed, one can overcome various barriers of nanoparticle delivery into tumors by several methods, e.g. by regulation of vessels, regulation of permeability, physical disruption of vessels, and modification of the tumor microenvironment through cancer associated fibroblasts [89, 90, 92]. Additionally, high genetic heterogeneity of cancer leads to an enormously high variability in the EPR effect [89, 90, 92].

**Fig. 8:**
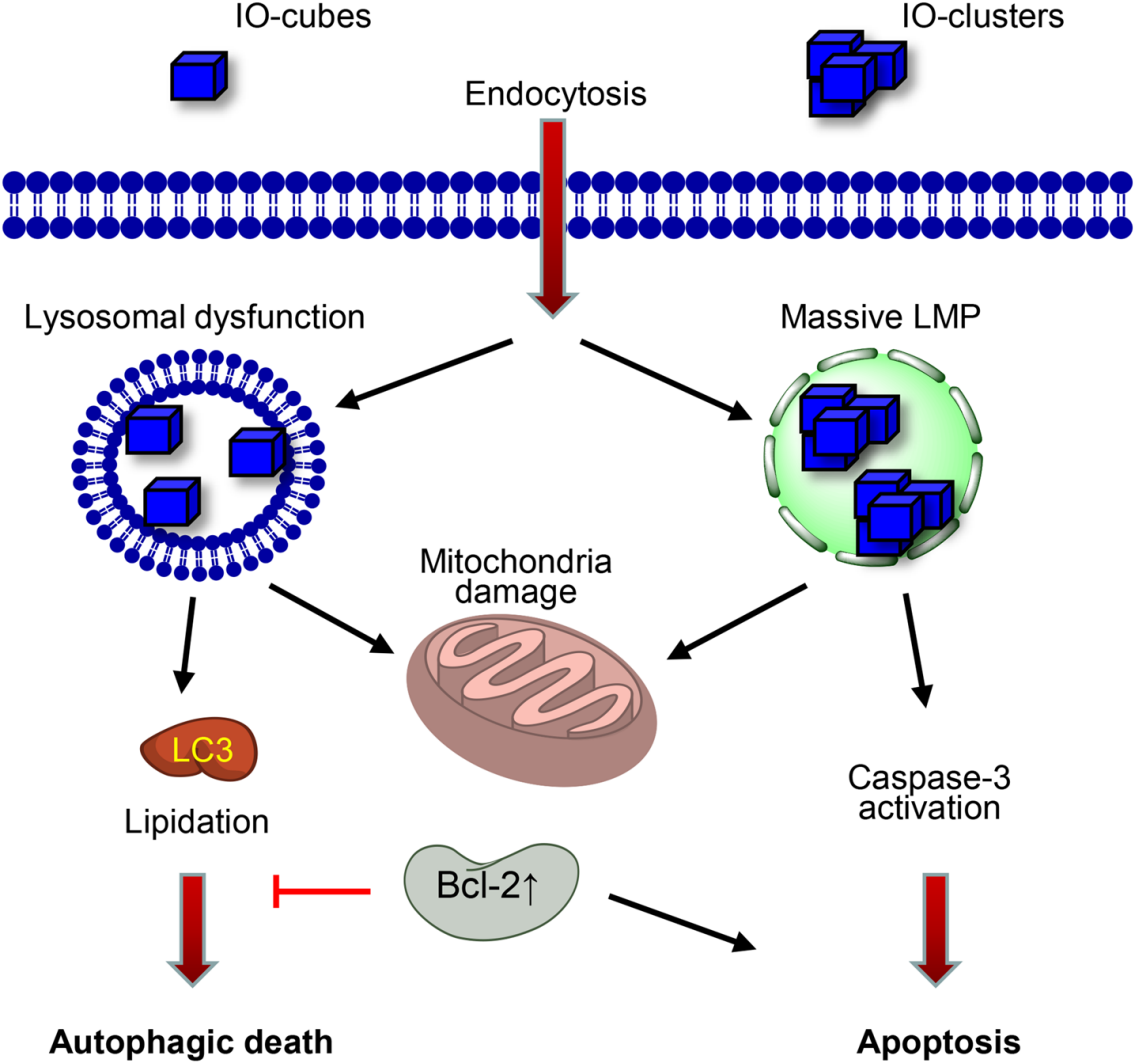
Scheme of cell signaling activation after stimulation with IO-cubes or IO-clusters. *LMP* lysosomal membrane permeabilization

However, we also found that genetic background is crucial for ION-mediated response in liver tumor cells. Whereas hepatoblastoma-derived HepG2 cells treated with either IO-cubes or IO-clusters underwent apoptotic death (Figs. 4, 5), treatment of Alexander and Huh7 representing hepatocellular carcinoma cells with IO-cubes led to autophagic death (Figs. 4, 5). However, IO-clusters triggered apoptosis in Alexander and Huh7 cells (Figs. 4, 5). We and other have previously shown that HepG2 cells express higher levels of Bcl-2 than Alexander and Huh7 cells [55, 83, 84]. In fact, Bcl-2 negatively regulates autophagy [85–87]. Given that there is a substantial molecular crosstalk between apoptosis and autophagic death pathway [51, 52], it becomes understandable why both nanoparticles induce rather apoptosis than autophagy in HepG2 cells. High Bcl-2 levels counteract autophagic flux in HepG2 treated with either IO-cubes or IO-clusters (Fig. 8). However, LMP induced by these NPs progressively aggravates with time and in turn results in apoptotic death (Fig. 8).

Taking into account challenges with targeted delivery of nanoparticles, their liver interaction and clearance represent an important issue. Recent studies estimate that 30–99% of administered nanoparticles are sequestered by the liver after administration into the body [68]. This may lead to increased liver cells toxicity (see [68] for review). Importantly, a recent study showed that in liver tissue nanoparticles are taken up and cleared by many cell types, including Kupffer cells, hepatic cells and endothelial cells, and not just by Kupffer cells as expected [93]. In typical liver microstructure, liver sinusoids have fenestrae that are holes of 50–200 nm in diameter [66]. Therefore, nanoparticles smaller than 200 nm in diameter can pass easily through these holes [66, 68]. Upon forced extrusion even particles of 400 nm in diameter can extravasate through the liver sinusoid [94]. These data imply that there is a need to elucidate in detail molecular basis of nanoparticle-liver cell interactions. Indeed, most in vitro studies are focused on a single hepatic cell type in culture and do not consider genetic variability among distinct cell lines [68]. Therefore, in our study we analyzed nanoparticle-liver cell responses utilizing three cell lines. Our data imply that genetic background of cells treated with nanoparticles greatly affects subsequent signaling.

In Alexander and Huh7 carcinoma cells IO-cubes and IO-clusters trigger different cell death scenarios. IO-clusters induced higher LMP level in comparison with IO-cubes in Alexander and Huh7 cells (Fig. 7e). Thus, massive damage to lysosomal membranes induced by IO-clusters led to execution of apoptosis (Figs. 4, 5). Contrary, LMP induced by IO-cubes was mild and resulted in autophagic cell death (Figs. 4, 5, 6, 7).

This study provides evidence that genetic background of cells treated with nanoparticles predisposes the outcomes due to different expression levels of Bcl-2. In fact, both nanoparticles induced rather apoptosis than autophagy in HepG2. Contrary, IO-cubes and IO-clusters trigger distinct cell death signalling events in Alexander and Huh7 cells.

## Conclusions

Present study reveals the mechanism by which cubic nanoparticles induce autophagic flux and the mechanism of subsequent toxicity. Our data indicate that the cytotoxic effects of iron oxide nanoparticles require more intensive study and that they should be considered in biomedical applications, particularly in patients with liver diseases.

## Supplementary information

**Additional file 1. Figure S1.** Transmission electron micrographs of the iron core of the nanoparticles. **Figure S2.** Annexin V-PI staining of Alexander cells. **Figure S3.** Annexin V-PI staining of Huh7 cells. **Figure S4.** Annexin V-PI staining of HepG2 cells. **Figure S5.** Alteration of mitochondrial morphology by IO-cubes and IO-clusters treatment. **Figure S6.** Colocalization of fluorescently labeled nanoparticles with lysosomes in Alexander cells. **Figure S7.** Colocalization of fluorescently labeled nanoparticles with lysosomes in Huh7 cells. **Figure S8.** Colocalization of fluorescently labeled nanoparticles with lysosomes in HepG2 cells. Uncropped immunoblot scans.

## Data Availability

The datasets used in this study are available from the corresponding author upon reasonable request.

## Abbreviations

IONs: Iron oxide nanoparticles
MRI: Magnetic resonance imaging
ROS: Reactive oxygen species
LMP: Lysosomal membrane permeabilization
NPs: Nanoparticles
