# Supplementary material for: Progressive lysosomal membrane permeabilization induced by iron oxide nanoparticles drives hepatic cell autophagy and apoptosis
Source: Nano Converg. 2020 May 19;7:17. doi: 10.1186/s40580-020-00228-5 (PMC7235155; doi:10.1186/s40580-020-00228-5)

Additional file

Progressive lysosomal membrane permeabilization induced by iron oxide nanoparticles drives hepatic cell autophagy and apoptosis

Kateryna Levada[1], Stanislav Pshenichnikov[1], Alexander Omelyanchik[1], Valeria Rodionova[1], Aleksey Nikitin[2], Alexander Savchenko[2], Igor Schetinin[2], Dmitry Zhukov[2], Maxim Abakumov[2], Alexander Majouga[2], Mariia Lunova[3,4], Milan Jirsa[4], Barbora Smolková[3], Mariia Uzhytchak[3], Alexandr Dejneka[3] and Oleg Lunov[3]*

[1]*Institute of Physics, Mathematics and Information Technology, Immanuel Kant Baltic Federal University, Kaliningrad, Russia*

[2]*National University of Science and Technology "MISIS", Moscow, Russia*

[3]*Institute of Physics of the Czech Academy of Sciences, Prague, Czech Republic*

[4]*Institute for Clinical & Experimental Medicine (IKEM), Prague, Czech Republic*

[*] Corresponding author at: Institute of Physics of the Czech Academy of Sciences, Prague, 18221, Czech Republic.

E-mail address: lunov@fzu.cz (O. Lunov).

Additional figures

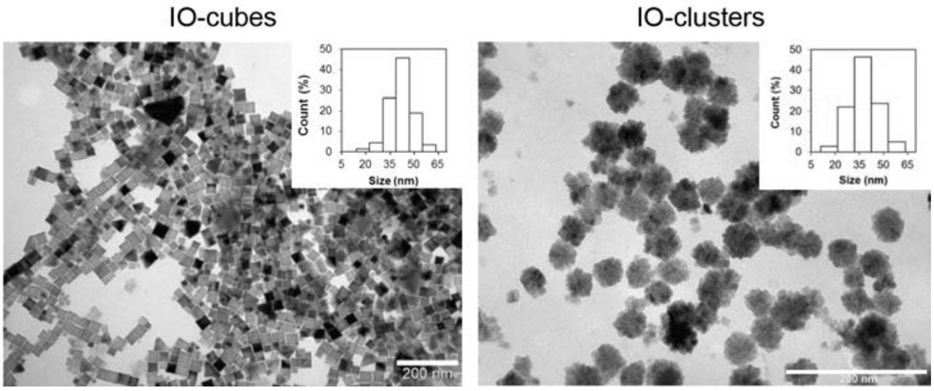

Figure S1. Transmission electron micrographs of the iron core of the nanoparticles.

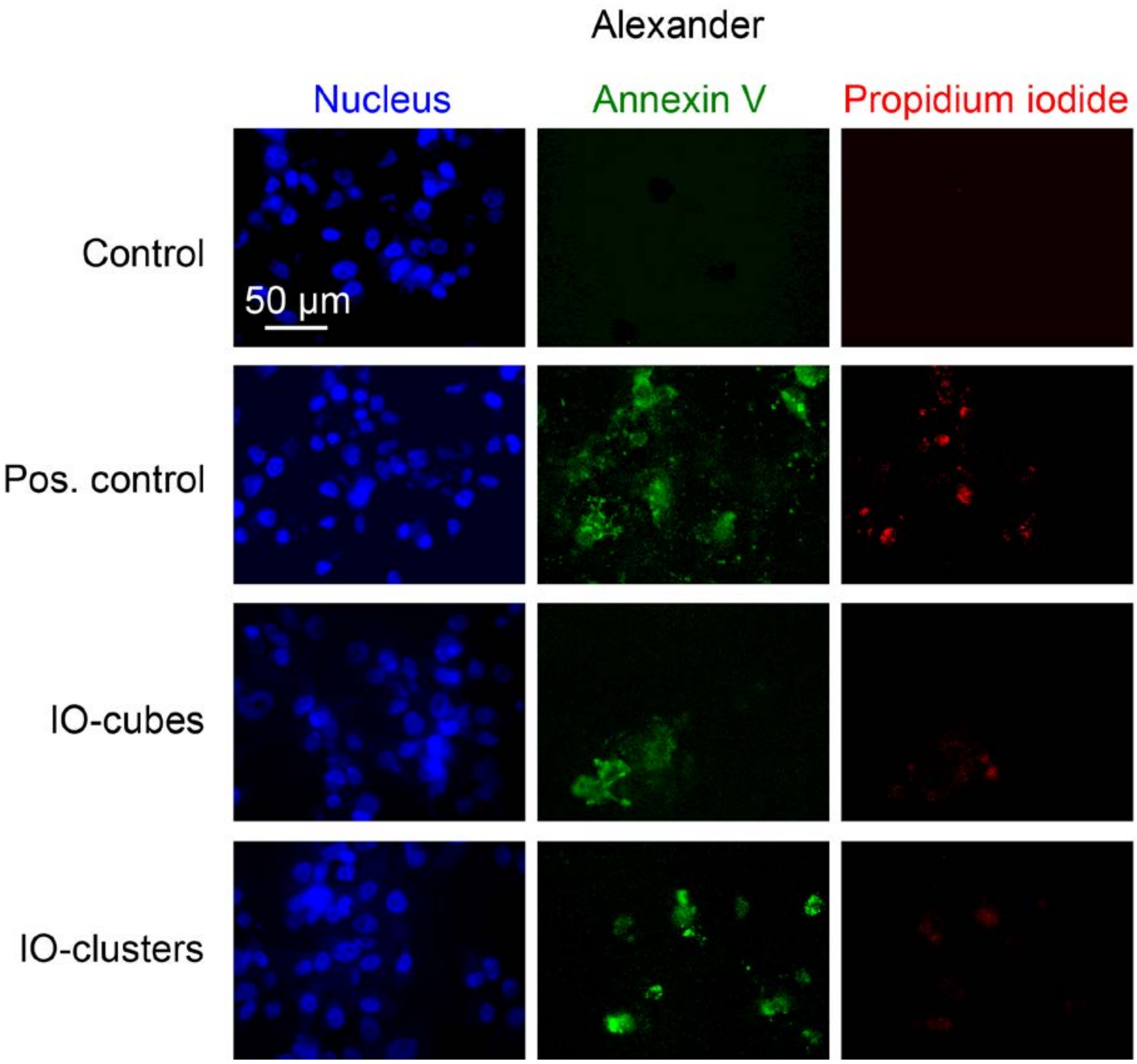

Figure S2. Alexander cells were stimulated with IO-cubes or IO-clusters (100 µg/mL) for 24 h and labeled with annexin V – green dye, propidium iodide – red dye and hoechst 33342 nuclear stain – blue. Labeled cells were imaged with epi-fluorescence microscopy.

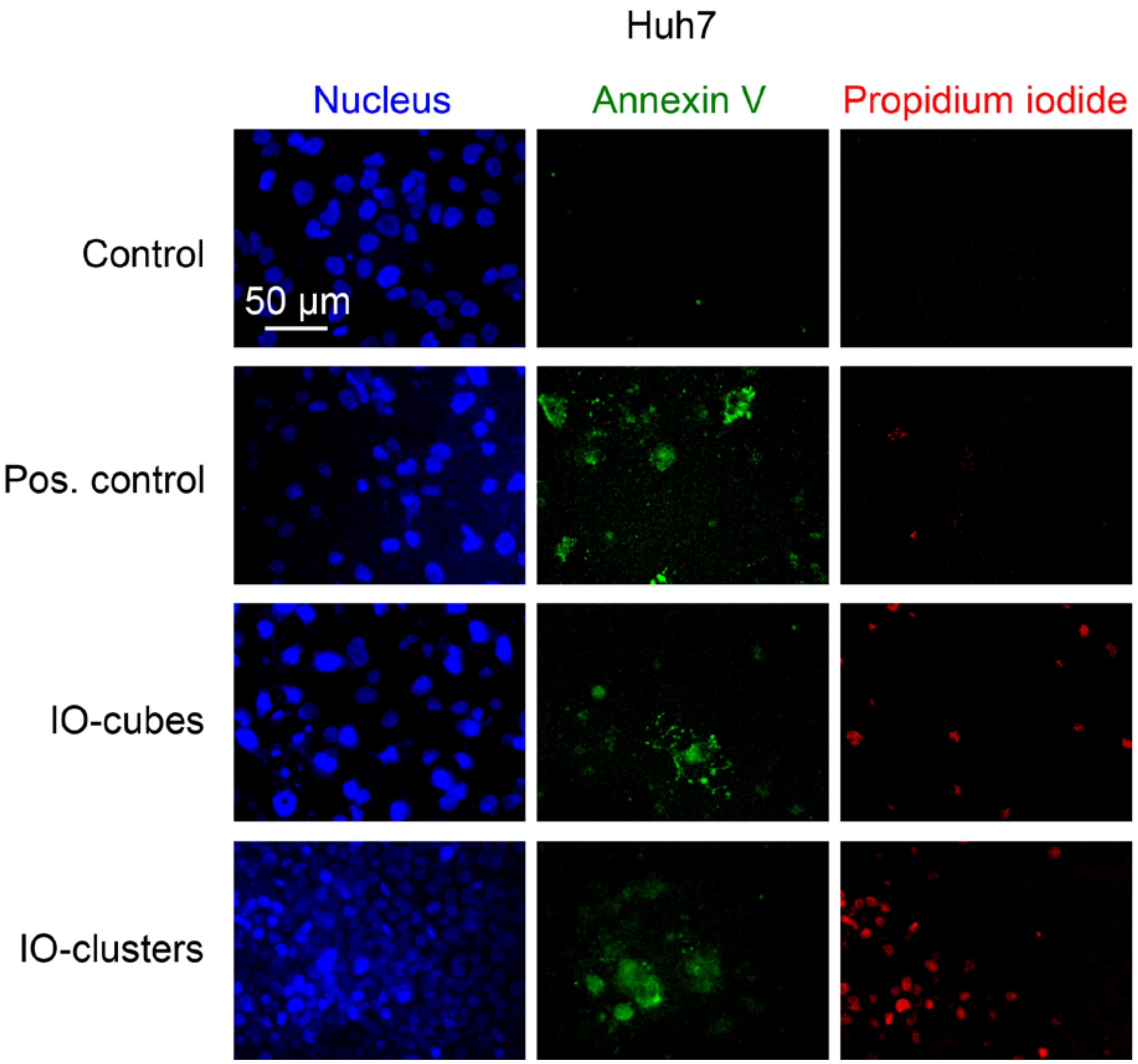

Figure S3. Huh7 cells were stimulated with IO-cubes or IO-clusters (100 µg/mL) for 24 h and labeled with annexin V – green dye, propidium iodide – red dye and hoechst 33342 nuclear stain – blue. Labeled cells were imaged with epi-fluorescence microscopy.

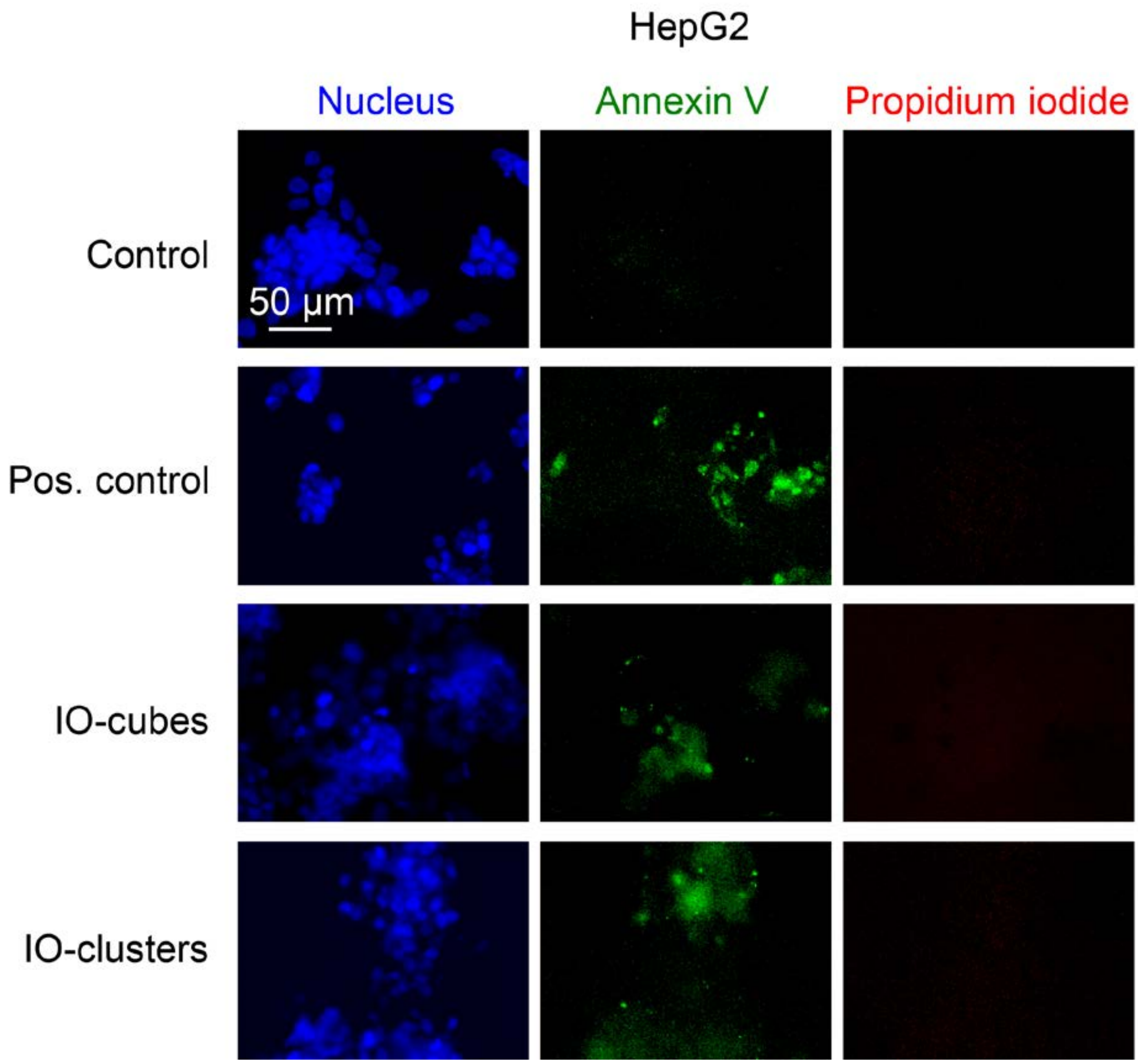

Figure S4. HepG2 cells were stimulated with IO-cubes or IO-clusters (100 µg/mL) for 24 h and labeled with annexin V – green dye, propidium iodide – red dye and hoechst 33342 nuclear stain – blue. Labeled cells were imaged with epi-fluorescence microscopy.

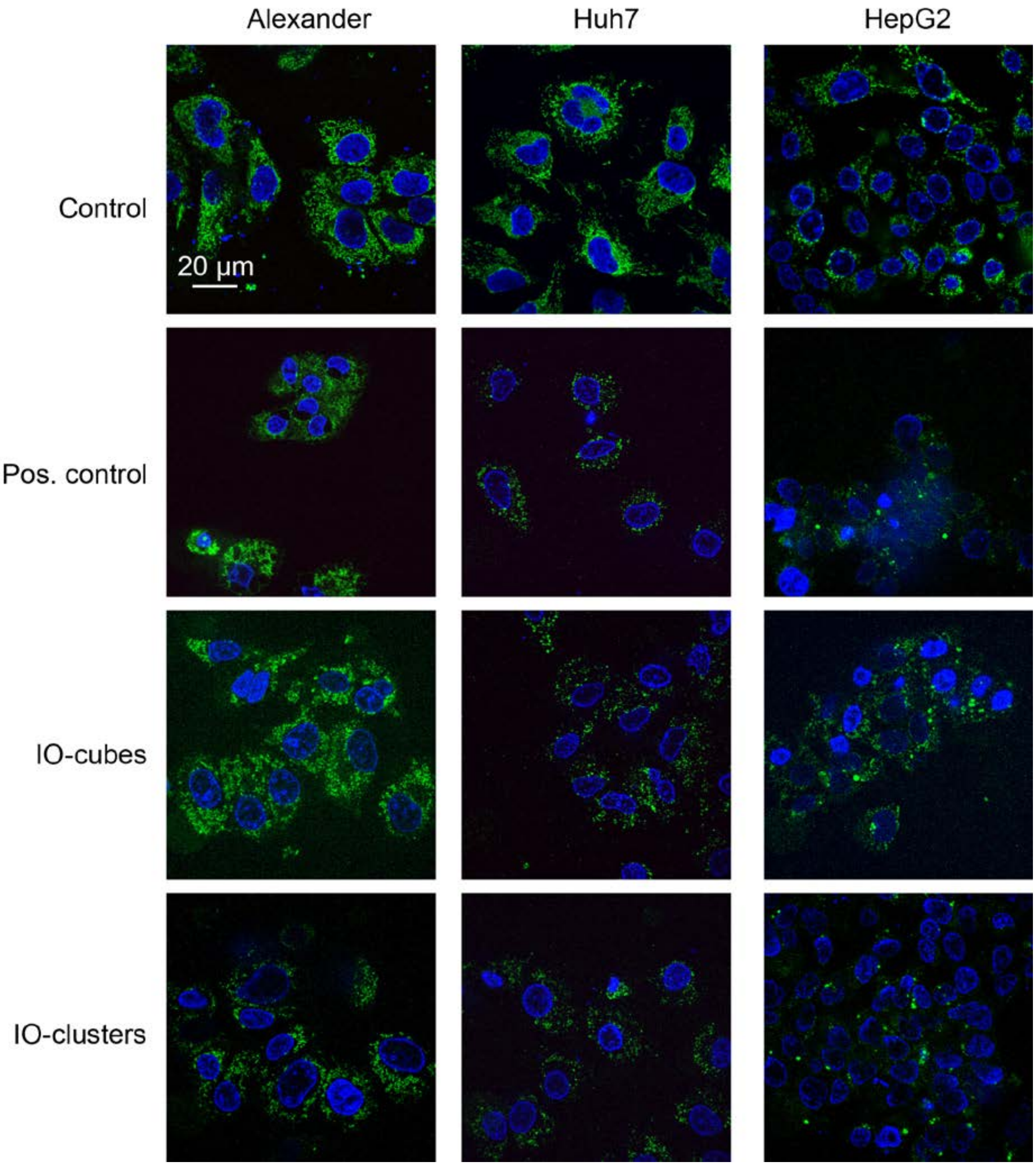

Figure S5. Alteration of mitochondrial morphology by IO-cubes and IO-clusters treatment. Alexander, HepG2 and Huh7 cells were stimulated with IO-cubes or IO-clusters (100 µg/mL) for 24 h and labeled with MitoTracker® green. Positive control – 20 % ethanol for 20 min. Nuclei were labelled with hoechst 33342 nuclear stain (blue). Labeled cells were then imaged using spinning disk confocal microscopy.

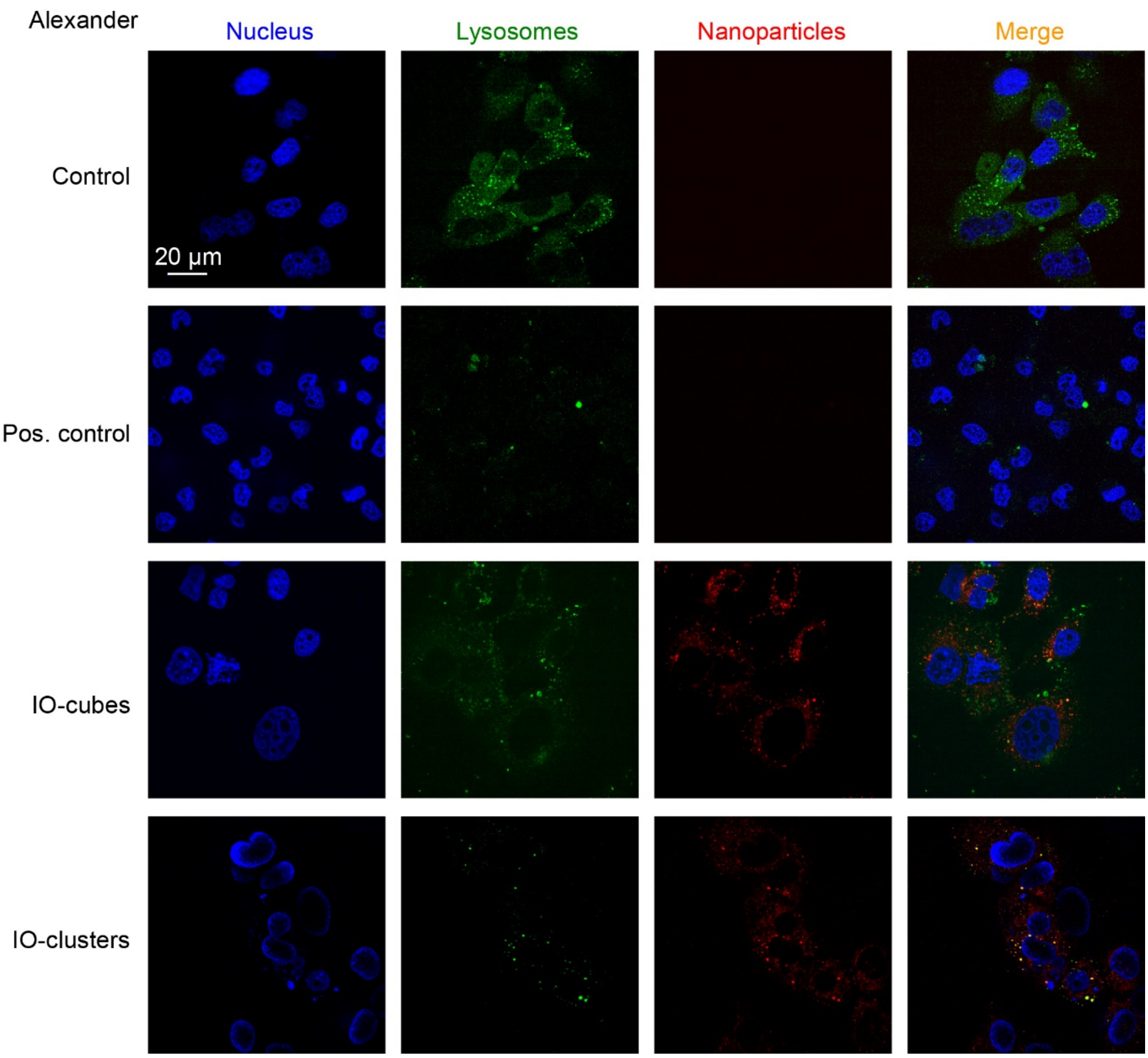

Figure S6. Alexander cells were treated with fluorescently labeled (red) IO-cubes or IO-clusters (100 µg/mL) for 24 h and stained with LysoTracker (green), colocalization of fluorescently labeled nanoparticles with lysosomes (yellow). Positive control – 20 % ethanol for 20 min. Nuclei were labelled with hoechst 33342 nuclear stain (blue). Labeled cells were then imaged using spinning disk confocal microscopy.

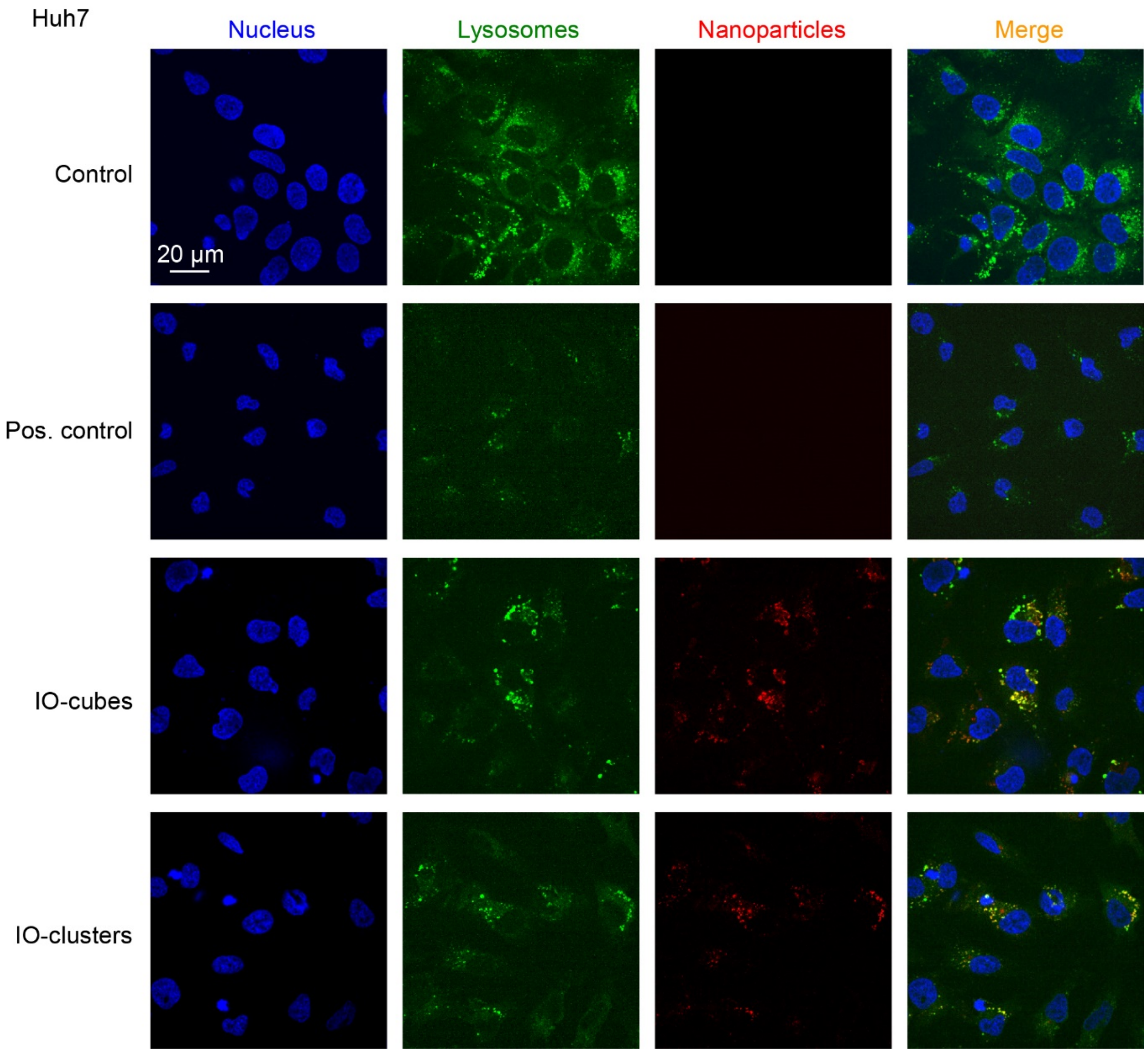

Figure S7. Huh7 cells were treated with fluorescently labeled (red) IO-cubes or IO-clusters (100 µg/mL) for 24 h and stained with LysoTracker (green), colocalization of fluorescently labeled nanoparticles with lysosomes (yellow). Positive control – 20 % ethanol for 20 min. Nuclei were labelled with hoechst 33342 nuclear stain (blue). Labeled cells were then imaged using spinning disk confocal microscopy.

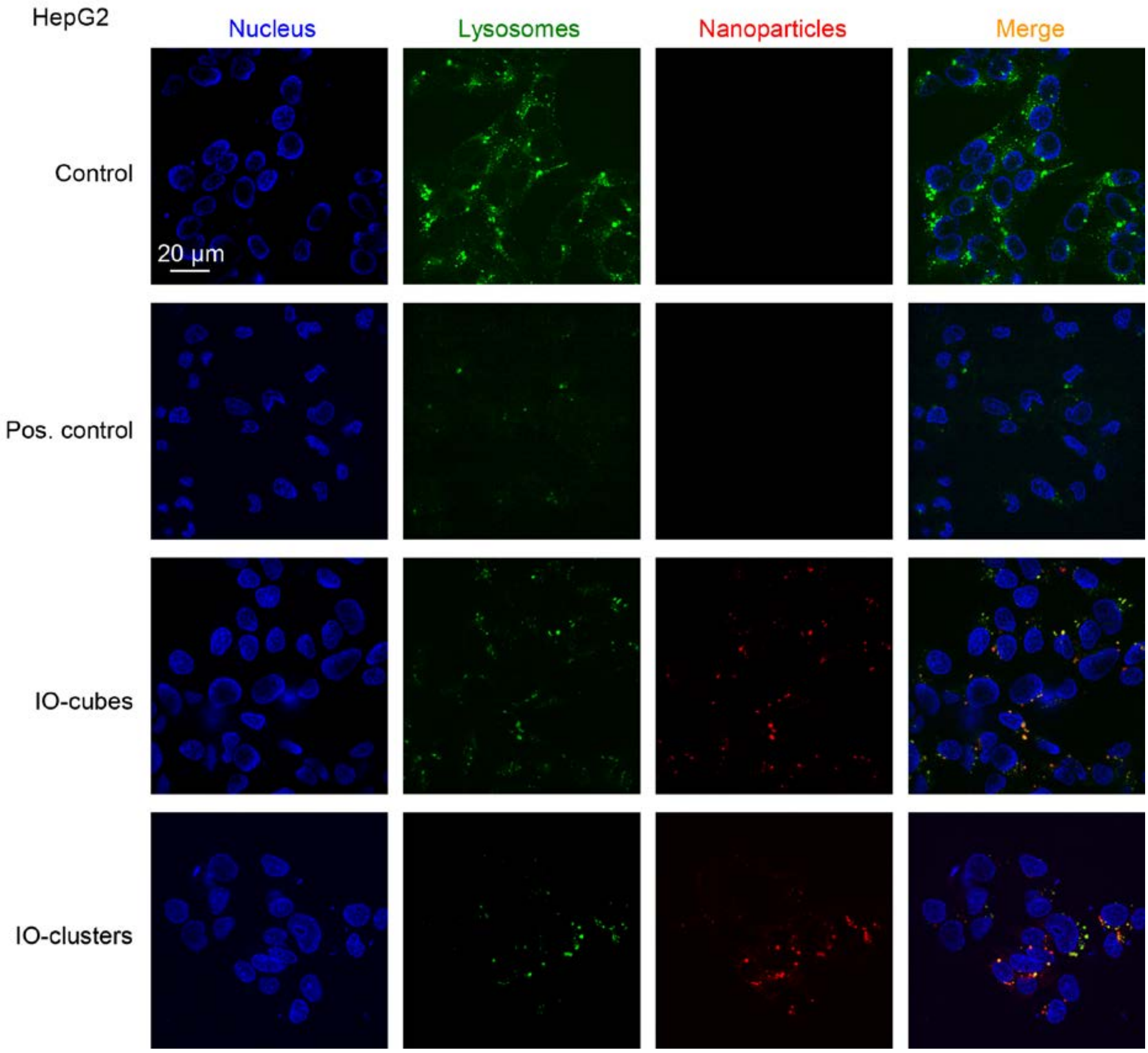

Figure S8. HepG2 cells were treated with fluorescently labeled (red) IO-cubes or IO-clusters (100 µg/mL) for 24 h and stained with LysoTracker (green), colocalization of fluorescently labeled nanoparticles with lysosomes (yellow). Positive control – 20 % ethanol for 20 min. Nuclei were labelled with hoechst 33342 nuclear stain (blue). Labeled cells were then imaged using spinning disk confocal microscopy.

Uncropped immunoblot scans

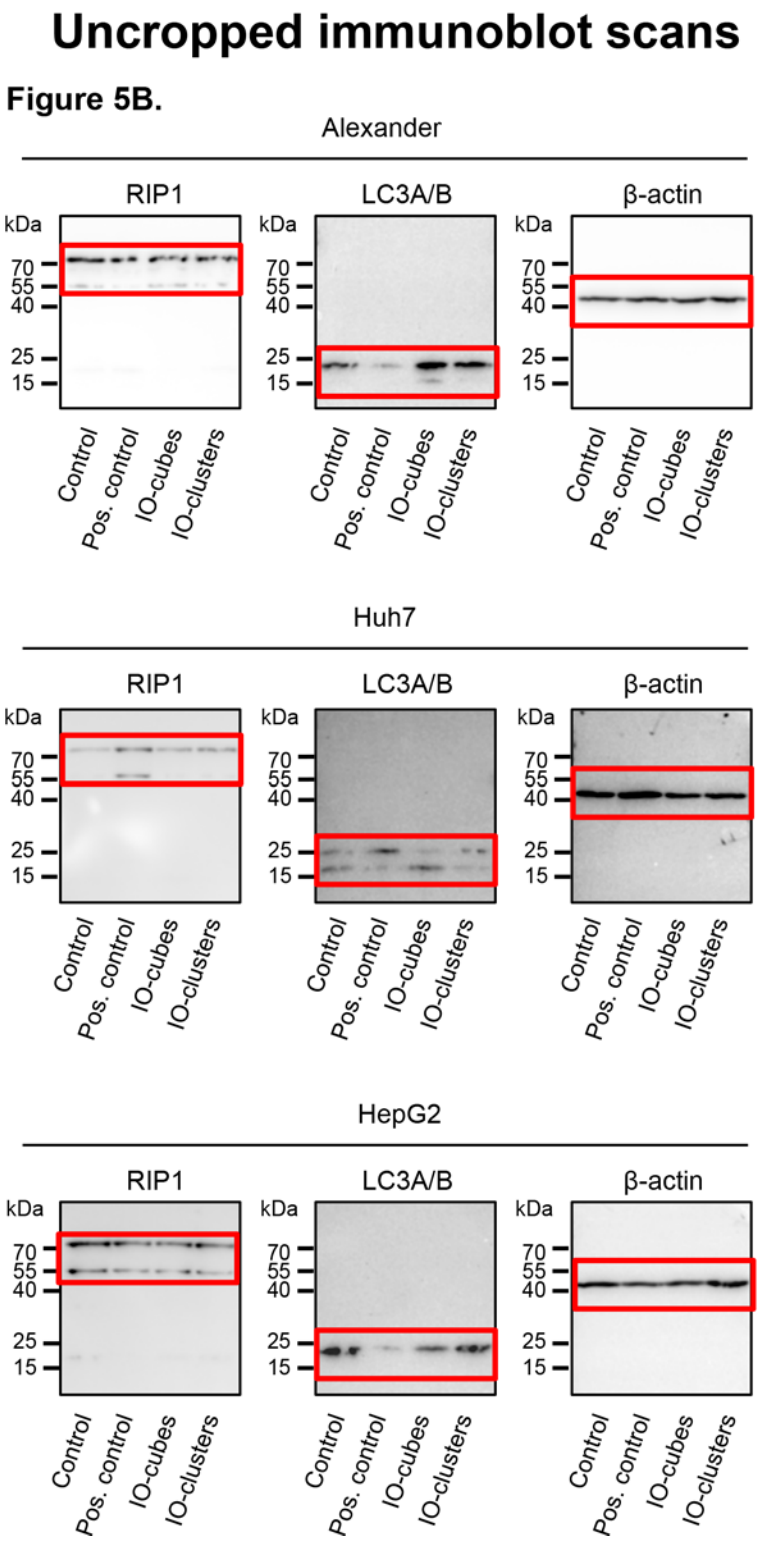

Supplement: Supplementary file 1 — Additional file 1. Figure S1. Transmission electron micrographs of the iron core of the nanoparticles. Figure S2. Annexin V-PI staining of Alexander cells. Figure S3. Annexin V-PI staining of Huh7 cells. Figure S4. Annexin V-PI staining of HepG2 cells. Figure S5. Alteration of mitochondrial morphology by IO-cubes and IO-clusters treatment. Figure S6. Colocalization of fluorescently labeled nanoparticles with lysosomes in Alexander cells. Figure S7. Colocalization of fluorescently labeled nanoparticles with lysosomes in Huh7 cells. Figure S8. Colocalization of fluorescently labeled nanoparticles with lysosomes in HepG2 cells. Uncropped immunoblot scans. [file 40580_2020_228_MOESM1_ESM.pdf]
